# Supplementary material for: Pilot study of Tremelimumab with and without cryoablation in patients with metastatic renal cell carcinoma
Source: Nat Commun. 2021 Nov 4;12:6375. doi: 10.1038/s41467-021-26415-4 (PMC8569213; doi:10.1038/s41467-021-26415-4)
Supplement: Supplementary file 1 — Supplementary Information [file 41467_2021_26415_MOESM1_ESM.pdf]

Pilot study of Tremelimumab with and without cryoablation in patients with metastatic renal cell carcinoma

Matthew T Campbell[1], Surena F Matin[2], Alda L Tam[3], Rahul A Sheth[3], Kamran Ahrar[3], Rebecca S Tidwell[4], Priya Rao[5]; Jose A Karam[2,6], Christopher G Wood[2], Nizar M Tannir[1], Eric Jonasch[1], Jianjun Gao[1], Amado J Zurita[1], Amishi Y Shah[1], Sonali Jindal[7], Fei Duan[7], Sreyashi Basu[7], Hong Chen[7], Alexsandra B Espejo[7], James P Allison[7,8], Shalini S Yadav[7], Padmanee Sharma[1,7,8*].

Author Affiliations:

[1]: Department of Genitourinary Medical Oncology, The University of Texas MD Anderson, Houston, TX
[2]: Department of Urology, The University of Texas MD Anderson Cancer Center, Houston, TX
[3]: Department of Interventional Radiology, The university of Texas of MD Anderson Cancer Center, Houston, TX
[4]: Department of Biostatistics, The University of Texas MD Anderson Cancer Center, Houston, TX
[5]: Department of Pathology, The University of Texas MD Anderson Cancer Center, Houston, TX
[6]: Department of Translational Molecular Pathology, The University of Texas MD Anderson Cancer Center, Houston, TX
[7]: The Immunotherapy Platform, The University of Texas MD Anderson Cancer Center, Houston, TX
[8]: Department of Immunology, The University of Texas MD Anderson Cancer Center, Houston, TX
[*]: Corresponding Author

Supplementary Fig. 1: Pathway analysis of pre-treatment tumor tissue samples of mRCC patients with clear cell (mccRCC) and non clear cell (mnccRCC) histology. Nanostring analysis comparing pre-treatment tissue samples of 20 mRCC patients with clear cell (mccRCC) versus non-clear cell (mnccRCC) histology (mccRCC, n=11 mnccRCC, n=9) Heatmap showing (a) *VEGF* signaling, (b) *IFN-γ* signaling and (c) Neutrophil degranulaltion pathways showing differences between pre-treatment tissue samples of mccRCC and mnccRCC patients.

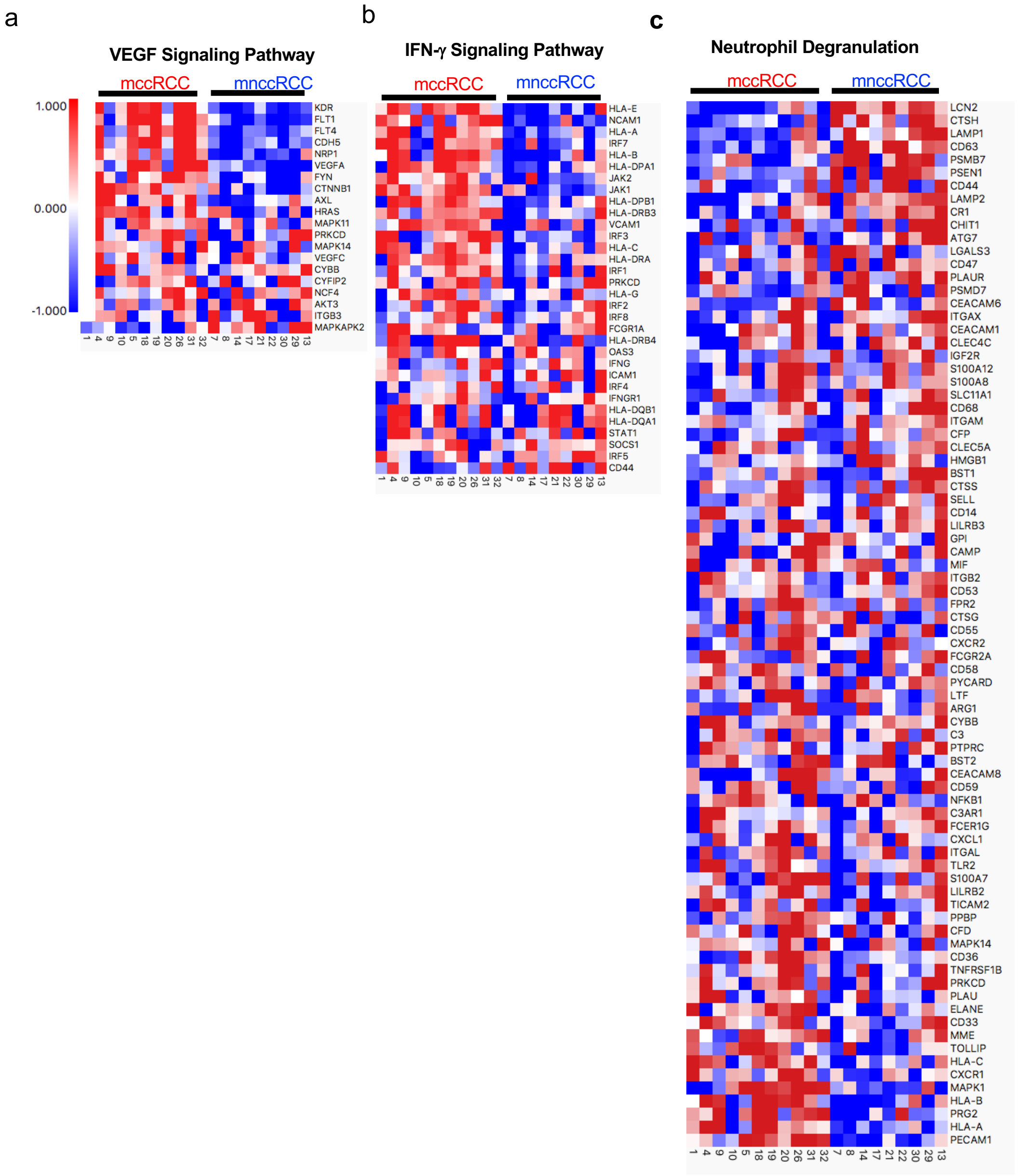

Supplementary Fig. 2: Analysis of tertiary lymphoid structures (TLS) in pre and post-treatment tissue samples of mRCC patients with clear-cell (mccRCC) and non clear cell (mnccRCC) histology. Pre and post-treatment tissue samples of patients with clear cell (mccRCC) and non-clear cell (mnccRCC) histology were analyzed by NanoString (mccRCC: pre (n=11), post (n=11); mnccRCC: pre (n=9), post (n=5)) Heatmap showing expression of TLS signature specific genes in pre and post-treatment tissue samples analyzed by NanoString.

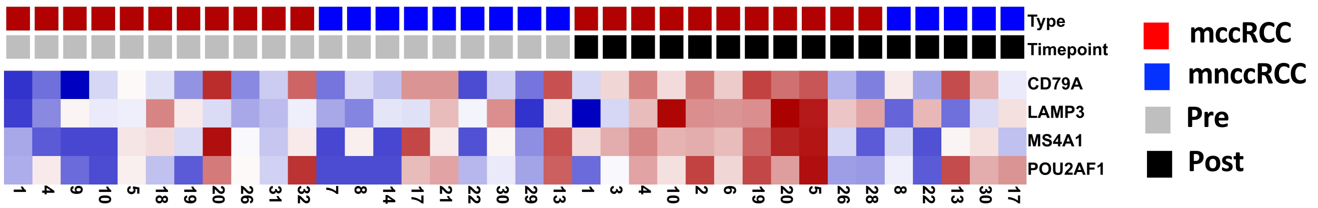

Supplementary Table 1: Number of Patients with Adverse Events by Treatment Arm Regardless of Attribution

	Treme Alone (N=14)		Cryo+Treme (N=15)		Total (N=29)	
	All	Grade 3+	All	Grade 3+	All	Grade 3+
Adverse Event	N (%)	N (%)	N (%)	N (%)	N (%)	N (%)
Laboratory	14 (100%)	2 (14%)	15 (100%)	4 (27%)	29 (100%)	6 (21%)
Colitis	2 (14%)	2 (14%)	3 (20%)	3 (20%)	5 (17%)	5 (17%)
Diarrhea	5 (36%)	1 (7%)	7 (47%)	3 (20%)	12 (41%)	4 (14%)
Rash	11 (79%)	1 (7%)	13 (87%)	2 (13%)	24 (83%)	3 (10%)
Pain	6 (43%)	1 (7%)	12 (80%)	2 (13%)	18 (62%)	3 (10%)
ALT increased	5 (36%)	2 (14%)	5 (33%)	1 (7%)	10 (34%)	3 (10%)
Nausea	3 (21%)	1 (7%)	10 (67%)	1 (7%)	13 (45%)	2 (7%)
Insomnia	3 (21%)	0 (0%)	7 (47%)	2 (13%)	10 (34%)	2 (7%)
Dyspnea	3 (21%)	2 (14%)	6 (40%)	0 (0%)	9 (31%)	2 (7%)
AST increased	3 (21%)	1 (7%)	5 (33%)	1 (7%)	8 (28%)	2 (7%)
Infection	4 (29%)	1 (7%)	4 (27%)	1 (7%)	8 (28%)	2 (7%)
Abdominal symptom	1 (7%)	0 (0%)	3 (20%)	2 (13%)	4 (14%)	2 (7%)
Pleural effusion	0 (0%)	0 (0%)	3 (20%)	2 (13%)	3 (10%)	2 (7%)
Autoimmune disorder	0 (0%)	0 (0%)	2 (13%)	2 (13%)	2 (7%)	2 (7%)
Fatigue	8 (57%)	1 (7%)	12 (80%)	0 (0%)	20 (69%)	1 (3%)
Cytopenia	7 (50%)	0 (0%)	8 (53%)	1 (7%)	15 (52%)	1 (3%)
Pruritus	6 (43%)	0 (0%)	7 (47%)	1 (7%)	13 (45%)	1 (3%)
Anorexia	2 (14%)	1 (7%)	6 (40%)	0 (0%)	8 (28%)	1 (3%)
Fever	5 (36%)	0 (0%)	2 (13%)	1 (7%)	7 (24%)	1 (3%)
Vomiting	2 (14%)	0 (0%)	4 (27%)	1 (7%)	6 (21%)	1 (3%)
Edema	1 (7%)	0 (0%)	3 (20%)	1 (7%)	4 (14%)	1 (3%)
Ocular	1 (7%)	0 (0%)	3 (20%)	1 (7%)	4 (14%)	1 (3%)
Paresthesia	1 (7%)	0 (0%)	3 (20%)	1 (7%)	4 (14%)	1 (3%)
Acute kidney injury	0 (0%)	0 (0%)	2 (13%)	1 (7%)	2 (7%)	1 (3%)
Dysphoric mood	1 (7%)	0 (0%)	1 (7%)	1 (7%)	2 (7%)	1 (3%)
Hyperthyroidism	1 (7%)	0 (0%)	1 (7%)	1 (7%)	2 (7%)	1 (3%)
Neoplasms benign, malignant and unspecified (incl cysts and polyps) - Other, specify	1 (7%)	0 (0%)	1 (7%)	1 (7%)	2 (7%)	1 (3%)
Hepatobiliary disorders - Other, specify-Hepatitis	1 (7%)	1 (7%)	0 (0%)	0 (0%)	1 (3%)	1 (3%)
Hypophysitis	1 (7%)	1 (7%)	0 (0%)	0 (0%)	1 (3%)	1 (3%)
Investigations - Other, specify-Decreased activity	1 (7%)	1 (7%)	0 (0%)	0 (0%)	1 (3%)	1 (3%)
Pleuritic pain	0 (0%)	0 (0%)	1 (7%)	1 (7%)	1 (3%)	1 (3%)
Pneumonitis	0 (0%)	0 (0%)	1 (7%)	1 (7%)	1 (3%)	1 (3%)
Watering eyes	1 (7%)	1 (7%)	0 (0%)	0 (0%)	1 (3%)	1 (3%)
Weakness	1 (7%)	1 (7%)	0 (0%)	0 (0%)	1 (3%)	1 (3%)
Constipation	5 (36%)	0 (0%)	6 (40%)	0 (0%)	11 (38%)	0 (0%)
Weight loss	4 (29%)	0 (0%)	7 (47%)	0 (0%)	11 (38%)	0 (0%)
Cough	5 (36%)	0 (0%)	5 (33%)	0 (0%)	10 (34%)	0 (0%)
Allergic rhinitis	4 (29%)	0 (0%)	4 (27%)	0 (0%)	8 (28%)	0 (0%)
Headache	4 (29%)	0 (0%)	4 (27%)	0 (0%)	8 (28%)	0 (0%)
Dizziness	1 (7%)	0 (0%)	6 (40%)	0 (0%)	7 (24%)	0 (0%)
Arthralgia	2 (14%)	0 (0%)	4 (27%)	0 (0%)	6 (21%)	0 (0%)
Anxiety	3 (21%)	0 (0%)	2 (13%)	0 (0%)	5 (17%)	0 (0%)
Dysgeusia	1 (7%)	0 (0%)	3 (20%)	0 (0%)	4 (14%)	0 (0%)
Flu like symptoms	3 (21%)	0 (0%)	1 (7%)	0 (0%)	4 (14%)	0 (0%)

Supplementary Table 1: Number of Patients with Adverse Events by Treatment Arm Regardless of Attribution Continued

	Treme Alone (N=14)		Cryo+Treme (N=15)		Total (N=29)	
	All	Grade 3+	All	Grade 3+	All	Grade 3+
Adverse Event	N (%)	N (%)	N (%)	N (%)	N (%)	N (%)
Hypothyroidism	1 (7%)	0 (0%)	3 (20%)	0 (0%)	4 (14%)	0 (0%)
Lower urinary tract symptoms	4 (29%)	0 (0%)	0 (0%)	0 (0%)	4 (14%)	0 (0%)
Malaise	1 (7%)	0 (0%)	3 (20%)	0 (0%)	4 (14%)	0 (0%)
Dysuria	0 (0%)	0 (0%)	3 (20%)	0 (0%)	3 (10%)	0 (0%)
Hypotension	1 (7%)	0 (0%)	2 (13%)	0 (0%)	3 (10%)	0 (0%)
Myalgia	1 (7%)	0 (0%)	2 (13%)	0 (0%)	3 (10%)	0 (0%)
Non-cardiac chest pain	0 (0%)	0 (0%)	3 (20%)	0 (0%)	3 (10%)	0 (0%)
Skin	3 (21%)	0 (0%)	0 (0%)	0 (0%)	3 (10%)	0 (0%)
Tremor	1 (7%)	0 (0%)	2 (13%)	0 (0%)	3 (10%)	0 (0%)
Dehydration	1 (7%)	0 (0%)	1 (7%)	0 (0%)	2 (7%)	0 (0%)
Dry mouth	0 (0%)	0 (0%)	2 (13%)	0 (0%)	2 (7%)	0 (0%)
Edema limbs	2 (14%)	0 (0%)	0 (0%)	0 (0%)	2 (7%)	0 (0%)
Fall	2 (14%)	0 (0%)	0 (0%)	0 (0%)	2 (7%)	0 (0%)
Gastroesophageal reflux disease	0 (0%)	0 (0%)	2 (13%)	0 (0%)	2 (7%)	0 (0%)
Hoarseness	1 (7%)	0 (0%)	1 (7%)	0 (0%)	2 (7%)	0 (0%)
Nasal congestion	0 (0%)	0 (0%)	2 (13%)	0 (0%)	2 (7%)	0 (0%)
Peripheral sensory neuropathy	0 (0%)	0 (0%)	2 (13%)	0 (0%)	2 (7%)	0 (0%)
Sore throat	0 (0%)	0 (0%)	2 (13%)	0 (0%)	2 (7%)	0 (0%)
Tinnitus	1 (7%)	0 (0%)	1 (7%)	0 (0%)	2 (7%)	0 (0%)
Adrenal insufficiency	0 (0%)	0 (0%)	1 (7%)	0 (0%)	1 (3%)	0 (0%)
Agitation	1 (7%)	0 (0%)	0 (0%)	0 (0%)	1 (3%)	0 (0%)
Athletes foot	1 (7%)	0 (0%)	0 (0%)	0 (0%)	1 (3%)	0 (0%)
Autoimmune glomerulonephritis	0 (0%)	0 (0%)	1 (7%)	0 (0%)	1 (3%)	0 (0%)
Chills	1 (7%)	0 (0%)	0 (0%)	0 (0%)	1 (3%)	0 (0%)
Depression	0 (0%)	0 (0%)	1 (7%)	0 (0%)	1 (3%)	0 (0%)
Diaphoresis	1 (7%)	0 (0%)	0 (0%)	0 (0%)	1 (3%)	0 (0%)
Dyspepsia	0 (0%)	0 (0%)	1 (7%)	0 (0%)	1 (3%)	0 (0%)
Ear pain	0 (0%)	0 (0%)	1 (7%)	0 (0%)	1 (3%)	0 (0%)
Epistaxis	1 (7%)	0 (0%)	0 (0%)	0 (0%)	1 (3%)	0 (0%)
Fever-low grade	1 (7%)	0 (0%)	0 (0%)	0 (0%)	1 (3%)	0 (0%)
Flatulence	1 (7%)	0 (0%)	0 (0%)	0 (0%)	1 (3%)	0 (0%)
GI change	1 (7%)	0 (0%)	0 (0%)	0 (0%)	1 (3%)	0 (0%)
Generalized muscle weakness	1 (7%)	0 (0%)	0 (0%)	0 (0%)	1 (3%)	0 (0%)
Hematuria	0 (0%)	0 (0%)	1 (7%)	0 (0%)	1 (3%)	0 (0%)
Hypertension	0 (0%)	0 (0%)	1 (7%)	0 (0%)	1 (3%)	0 (0%)
Hypoxemia	0 (0%)	0 (0%)	1 (7%)	0 (0%)	1 (3%)	0 (0%)

Supplementary Table 1: Number of Patients with Adverse Events by Treatment Arm Regardless of Attribution Continued

	Treme Alone (N=14)		Cryo+Treme (N=15)		Total (N=29)	
	All	Grade 3+	All	Grade 3+	All	Grade 3+
Adverse Event	N (%)	N (%)	N (%)	N (%)	N (%)	N (%)
Neck pain	1 (7%)	0 (0%)	0 (0%)	0 (0%)	1 (3%)	0 (0%)
Palmar-plantar erythrodysesthesia syndrome	0 (0%)	0 (0%)	1 (7%)	0 (0%)	1 (3%)	0 (0%)
Postnasal drip	0 (0%)	0 (0%)	1 (7%)	0 (0%)	1 (3%)	0 (0%)
Presyncope	1 (7%)	0 (0%)	0 (0%)	0 (0%)	1 (3%)	0 (0%)
Rectal hemorrhage	0 (0%)	0 (0%)	1 (7%)	0 (0%)	1 (3%)	0 (0%)
Rhinorrhea	0 (0%)	0 (0%)	1 (7%)	0 (0%)	1 (3%)	0 (0%)
Scrotal pain	0 (0%)	0 (0%)	1 (7%)	0 (0%)	1 (3%)	0 (0%)
Sinus tachycardia	0 (0%)	0 (0%)	1 (7%)	0 (0%)	1 (3%)	0 (0%)
Tumor pain	0 (0%)	0 (0%)	1 (7%)	0 (0%)	1 (3%)	0 (0%)
Wheezing	1 (7%)	0 (0%)	0 (0%)	0 (0%)	1 (3%)	0 (0%)
Wrist fracture	1 (7%)	0 (0%)	0 (0%)	0 (0%)	1 (3%)	0 (0%)

Laboratory events include: Alkaline phosphatase increased, Blood bilirubin increased, Creatinine increased, Hypercalcemia, Hyperglycemia, Hyperkalemia, Hypoalbuminemia, Hypocalcemia, Hypokalemia, Hypomagnesemia, Hyponatremia, Hypophosphatemia, A1C elevated, ACTH decreased, ACTH elevated, ACTH increased, ANC elevated, Albumin elevated, Alk Phos elevated, Alkaline Phosphatase Increased (Bone), Amylase decreased, BUN elevated, Free T4 decreased, LDH elevated, Neutrophil count elevated, Phosphorus elevated, Platelets elevated, Sed rate, TSH, TSH elevated, Total protein elevated, WBC elevated, hyperphosphatemia, thyroid peroxidase antibody increased, Lipase increased, Ionized calcium decrease, phosphorus increased, Proteinuria, and Serum amylase increased.

Supplementary Table 2: Number of Patients with Related Adverse Events by Treatment Arm

	Treme Alone (N=14)		Cryo+Treme (N=15)		Total (N=29)	
	All	Grade 3+	All	Grade 3+	All	Grade 3+
Adverse Event	N (%)	N (%)	N (%)	N (%)	N (%)	N (%)
Laboratory	14 (100%)	2 (14%)	10 (67%)	4 (27%)	24 (83%)	6 (21%)
Colitis	2 (14%)	2 (14%)	3 (20%)	3 (20%)	5 (17%)	5 (17%)
Rash	10 (71%)	1 (7%)	11 (73%)	2 (13%)	21 (72%)	3 (10%)
ALT increased	5 (36%)	2 (14%)	3 (20%)	1 (7%)	8 (28%)	3 (10%)
Diarrhea	1 (7%)	0 (0%)	6 (40%)	3 (20%)	7 (24%)	3 (10%)
AST increased	3 (21%)	1 (7%)	1 (7%)	1 (7%)	4 (14%)	2 (7%)
Autoimmune disorder	0 (0%)	0 (0%)	2 (13%)	2 (13%)	2 (7%)	2 (7%)
Fatigue	5 (36%)	1 (7%)	9 (60%)	0 (0%)	14 (48%)	1 (3%)
Pruritus	6 (43%)	0 (0%)	7 (47%)	1 (7%)	13 (45%)	1 (3%)
Cytopenia	5 (36%)	0 (0%)	5 (33%)	1 (7%)	10 (34%)	1 (3%)
Nausea	2 (14%)	0 (0%)	5 (33%)	1 (7%)	7 (24%)	1 (3%)
Insomnia	2 (14%)	0 (0%)	3 (20%)	1 (7%)	5 (17%)	1 (3%)
Abdominal symptom	1 (7%)	0 (0%)	2 (13%)	1 (7%)	3 (10%)	1 (3%)
Anorexia	1 (7%)	1 (7%)	2 (13%)	0 (0%)	3 (10%)	1 (3%)
Fever	2 (14%)	0 (0%)	1 (7%)	1 (7%)	3 (10%)	1 (3%)
Vomiting	1 (7%)	0 (0%)	2 (13%)	1 (7%)	3 (10%)	1 (3%)
Edema	0 (0%)	0 (0%)	2 (13%)	1 (7%)	2 (7%)	1 (3%)
Hyperthyroidism	1 (7%)	0 (0%)	1 (7%)	1 (7%)	2 (7%)	1 (3%)
Acute kidney injury	0 (0%)	0 (0%)	1 (7%)	1 (7%)	1 (3%)	1 (3%)
Hepatobiliary disorders - Other, specify-Hepatitis	1 (7%)	1 (7%)	0 (0%)	0 (0%)	1 (3%)	1 (3%)
Hypophysitis	1 (7%)	1 (7%)	0 (0%)	0 (0%)	1 (3%)	1 (3%)
Investigations - Other, specify-Decreased activity	1 (7%)	1 (7%)	0 (0%)	0 (0%)	1 (3%)	1 (3%)
Pleuritic pain	0 (0%)	0 (0%)	1 (7%)	1 (7%)	1 (3%)	1 (3%)
Pneumonitis	0 (0%)	0 (0%)	1 (7%)	1 (7%)	1 (3%)	1 (3%)
Cough	2 (14%)	0 (0%)	3 (20%)	0 (0%)	5 (17%)	0 (0%)
Dyspnea	1 (7%)	0 (0%)	4 (27%)	0 (0%)	5 (17%)	0 (0%)
Headache	2 (14%)	0 (0%)	3 (20%)	0 (0%)	5 (17%)	0 (0%)
Pain	1 (7%)	0 (0%)	4 (27%)	0 (0%)	5 (17%)	0 (0%)
Dizziness	1 (7%)	0 (0%)	3 (20%)	0 (0%)	4 (14%)	0 (0%)
Hypothyroidism	1 (7%)	0 (0%)	3 (20%)	0 (0%)	4 (14%)	0 (0%)
Malaise	1 (7%)	0 (0%)	3 (20%)	0 (0%)	4 (14%)	0 (0%)
Weight loss	3 (21%)	0 (0%)	1 (7%)	0 (0%)	4 (14%)	0 (0%)
Hypotension	1 (7%)	0 (0%)	2 (13%)	0 (0%)	3 (10%)	0 (0%)
Ocular	1 (7%)	0 (0%)	2 (13%)	0 (0%)	3 (10%)	0 (0%)
Paresthesia	1 (7%)	0 (0%)	2 (13%)	0 (0%)	3 (10%)	0 (0%)
Arthralgia	1 (7%)	0 (0%)	1 (7%)	0 (0%)	2 (7%)	0 (0%)
Constipation	2 (14%)	0 (0%)	0 (0%)	0 (0%)	2 (7%)	0 (0%)
Myalgia	1 (7%)	0 (0%)	1 (7%)	0 (0%)	2 (7%)	0 (0%)
Adrenal insufficiency	0 (0%)	0 (0%)	1 (7%)	0 (0%)	1 (3%)	0 (0%)
Anxiety	0 (0%)	0 (0%)	1 (7%)	0 (0%)	1 (3%)	0 (0%)
Autoimmune glomerulonephritis	0 (0%)	0 (0%)	1 (7%)	0 (0%)	1 (3%)	0 (0%)

Supplementary Table 2: Number of Patients with Related Adverse Events by Treatment Arm Continued

Adverse Event	Treme Alone (N=14) All N (%)	Treme Alone (N=14) Grade 3+ N (%)	Cryo+Treme (N=15) All N (%)	Cryo+Treme (N=15) Grade 3+ N (%)	Total (N=29) All N (%)	Total (N=29) Grade 3+ N (%)
Dehydration	0 (0%)	0 (0%)	1 (7%)	0 (0%)	1 (3%)	0 (0%)
Dry mouth	0 (0%)	0 (0%)	1 (7%)	0 (0%)	1 (3%)	0 (0%)
Dysgeusia	0 (0%)	0 (0%)	1 (7%)	0 (0%)	1 (3%)	0 (0%)
Dyspepsia	0 (0%)	0 (0%)	1 (7%)	0 (0%)	1 (3%)	0 (0%)
Edema limbs	1 (7%)	0 (0%)	0 (0%)	0 (0%)	1 (3%)	0 (0%)
Flatulence	1 (7%)	0 (0%)	0 (0%)	0 (0%)	1 (3%)	0 (0%)
GI change	1 (7%)	0 (0%)	0 (0%)	0 (0%)	1 (3%)	0 (0%)
Gastroesophageal reflux disease	0 (0%)	0 (0%)	1 (7%)	0 (0%)	1 (3%)	0 (0%)
Hypertension	0 (0%)	0 (0%)	1 (7%)	0 (0%)	1 (3%)	0 (0%)
Hypoxemia	0 (0%)	0 (0%)	1 (7%)	0 (0%)	1 (3%)	0 (0%)
Infection	1 (7%)	0 (0%)	0 (0%)	0 (0%)	1 (3%)	0 (0%)
Neck pain	1 (7%)	0 (0%)	0 (0%)	0 (0%)	1 (3%)	0 (0%)
Palmar-plantar erythrodysesthesia syndrome	0 (0%)	0 (0%)	1 (7%)	0 (0%)	1 (3%)	0 (0%)
Peripheral sensory neuropathy	0 (0%)	0 (0%)	1 (7%)	0 (0%)	1 (3%)	0 (0%)
Pleural effusion	0 (0%)	0 (0%)	1 (7%)	0 (0%)	1 (3%)	0 (0%)
Skin	1 (7%)	0 (0%)	0 (0%)	0 (0%)	1 (3%)	0 (0%)
Tinnitus	0 (0%)	0 (0%)	1 (7%)	0 (0%)	1 (3%)	0 (0%)
Tremor	1 (7%)	0 (0%)	0 (0%)	0 (0%)	1 (3%)	0 (0%)
Wheezing	1 (7%)	0 (0%)	0 (0%)	0 (0%)	1 (3%)	0 (0%)

Supplementary Table 3: Baseline and on protocol tissue collection

	Arm A Tremelimumab monotherapy	Arm B Cryo+tremelimumab combination therapy
Baseline Biopsy	13/14	15/15
Pathology on Treatment	Surgery<br>ypT1aNX (clear cell)<br>ymp T1aNX (clear cell)<br>ypT3aN0M1 (clear cell)<br>ypT3aN1M1 (papillary)<br>ypT3aN2M1 (papillary)<br>metastasectomy	Surgery<br>ypT3aN0 (clear cell)<br>ypT3aN1 (clear cell)<br>ypT3aN2M1 (clear cell)<br>ypT3aN1M1 (papillary)<br>metastasectomy (clear cell)
Repeat biopsy without surgery	5	4
No on treatment Tissue	4	6

Cryo - Cryoablation

Supplementary Table 4: Tissue Samples analyzed by IHC and NanoString

mRCC histological subtype	IHC Pre-treatment	IHC Post-treatment	NanoString Pre-treatment	NanoString Post-treatment
Clear Cell (mccRCC)	13	11	11	11
Non-clear cell (mnccRCC)	11	5	9	5

mRCC – metastatic renal cell carcinoma, IHC – immunohistochemistry, mccRCC – metastatic clear cell renal cell carcinoma, mnccRCC – metastatic non clear cell renal cell carcinoma,

Supplementary Note 1: Study Protocol

MD Anderson IND Sponsor Cover Sheet	
**Protocol ID**	2013-0539
**Protocol Title**	PILOT STUDY OF TREMELIMUMAB WITH OR WITHOUT TISSUE CRYOABLATION IN PATIENTS WITH METASTATIC RENAL CELL CARCINOMA
**Protocol Phase**	Pilot
**Protocol Version**	14
**Version Date**	06/26/2018
**Protocol PI**	Padmanee Sharma MD, PhD
**Department**	Professor<br>Department of Genitourinary Medical Oncology<br>University of Texas, MD Anderson Cancer Center<br>1155 Pressler, Unit 1374<br>Houston, TX 77030
**Co-PI**	Surena F. Matin, MD
	Professor<br>Department of Urology, Unit 1373<br>University of Texas, MD Anderson Cancer Center<br>1515 Holcombe Blvd<br>Houston, TX 77030
**Co-PI**	Matthew Campbell, MD
	Assistant Professor<br>Department of Genitourinary Medical Oncology<br>University of Texas, MD Anderson Cancer Center<br>1155 Pressler, Unit 1374<br>Houston, TX 77030
**IND Sponsor**	MD Anderson Cancer Center
**IND #**	130,932
**Investigational Drug**	Durvalumab (MEDI4736) and Tremelimumab
**Study Number**	ESR-15-11590

Protocol v00-v02	Header 03/04/2014
Protocol v03	Header 04/18/2014
Protocol v04	Header 04/18/2014
Protocol v05-v06	Header 11/11/2015
Protocol v06	Header 06/27/2016
Protocol v07 -v09	Header 11/18/2016
Protocol v10	Header 03/15/2017
Protocol v11	Header 06/01/2017
Protocol v12	Header 06/01/2017
Protocol v13	Header 01/31/2018
Protocol v14	Header 06/26/2018

Study Outline

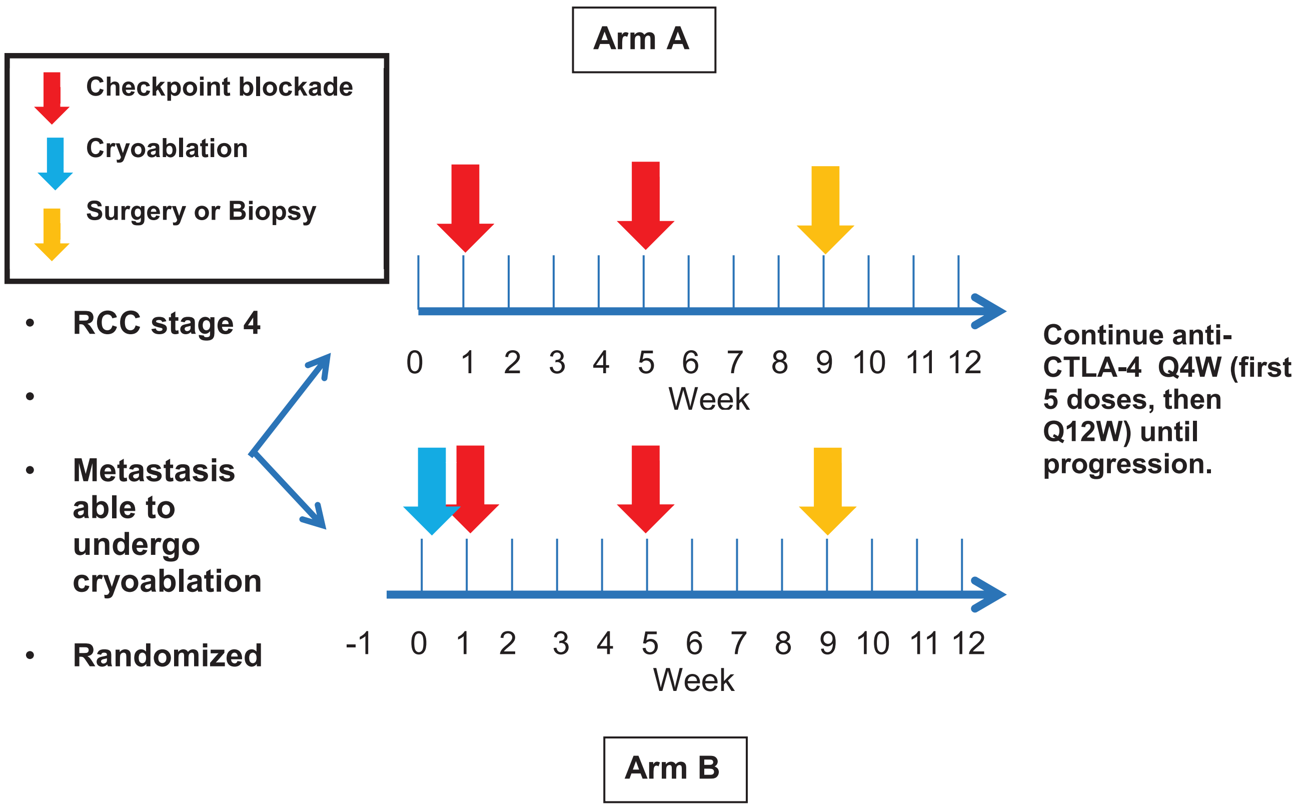

1.0 Objectives

1.1 Primary Objective:

- To evaluate ***safety*** of the tremelimumab alone and in combination with cryoablation in patients with metastatic RCC.

1.2 Secondary Objectives:

- To explore the longitudinal values for ***immunological variables*** (including CD4+ ICOS+ T cells; effector to regulatory T cell ratio; absolute lymphocyte count) following treatment with tremelimumab in patients with metastatic RCC, and evaluate difference between combination cryoablation and tremelimumab in comparison to tremelimumab alone.
- To determine the ***objective response rate*** and ***progression-free survival*** of patients treated with tremelimumab with or without cryoablation.

Hypothesis: The combination of cryoablation and tremelimumab therapy will be well tolerated in patients with metastatic RCC

2.0 Background

Anti-CTLA-4 therapy: T cell activation is a complex process that is initiated when an antigen is presented to the T cell receptor (TCR) followed by the interaction of additional T cell surface molecules with their respective ligands on the antigen presenting cell (APC). This second interaction can result in a positive or a negative costimulatory signal depending on which specific molecules are involved. CTLA-4 is a T cell co-inhibitory and immune checkpoint molecule expressed by T cells[1-4] that plays a critical role in maintaining immune homeostasis and peripheral tolerance to self-antigens.[5, 6] Several preclinical studies demonstrated that CTLA-4 blockade could augment T cell-mediated immune responses against tumors.[7, 8] This led to the development of ipilimumab, a fully humanized IgG_1 monoclonal antibody to CTLA-4. Two phase 3 trials demonstrated a durable clinical benefit for ~20-25% of metastatic melanoma patients with ipilimumab;[9, 10] resulting in the FDA approval of ipilimumab as a first- and second-line agent in metastatic melanoma. Tremelimumab is a similar compound as ipilimumab, extensively studied and currently in phase 3 trials, with a longer half-life [11-15]. Whichever is used, it is clear that anti-CTLA-4 therapy is effective as monotherapy in only a subset of patients with advanced disease.[9, 10] A phase I study explored tremelimumab in combination with sunitinib finding a high rate of dose limiting toxicity with tremelimumab in doses greater than 6mg/kg limiting further exploration of this combination.[16] Our goal is to develop approaches by which the effect can be augmented in the majority of patients by creating a potent initiator of immune response that will leverage the mechanism of tremelimumab.

Renal cell carcinoma (RCC): RCC has one of the highest mortalities of all genitourinary cancers with an approximate 25% death rate [17]. Despite these data, there is an increasing rate of detection in lower stage disease, and as well there has been a paradigm shift in treatment to more minimally invasive and kidney sparing treatments, even in those with

metastatic disease (mRCC) [18,19]. The initial standard for treatment of mRCC remains cytoreductive nephrectomy. The acceptance of surgical resection for the treatment of primary and metastatic tumors, has paved the way for cryoablation as an alternative for not only localized disease, but as well metastatic disease for treatment of both primary and metastatic sites [19-25]. The advent of targeted anti-vascular therapies has for the first time increased the survival of patients with metastatic RCC (mRCC) to over 2 year[26-]. Nevertheless, complete remissions (CRs) are very rare with these therapies, while with immune therapy a small but quantifiable proportion of patients experience long term CRs.

Cryoablation: Cryoablation and radiofrequency ablation (RFA) are the two most utilized and accepted forms of energy ablation for soft tissue and bone tumors. These two technologies can be applied to various soft tissues including kidney, liver, lung, bone, adrenal and other soft tissue areas, which incidentally happen to be the primary sites of metastasis for RCC. Ablative therapies are unique in that they result in the destruction of tumor in-situ and generate an intense inflammatory reaction and immune response [27]. The resulting necrotic tumor tissue is therefore exposed to an immunologic milieu highly favorable for induction of tumor-specific responses which can be unleashed by the addition of anti-CTLA-4 therapy. Our preliminary data suggests that local ablative therapy can lead to robust APC and T cell responses within tumor tissues. Additionally, our prior work in an orthotopic RCC murine model has shown a significant immune response with infiltration of neutrophils, macrophages, dendritic cells, CD4, and CD8 T cells after renal cryoablation of orthotopically implanted tumors [27]. There is a body of literature suggesting that in some instances the immunologic response after ablative therapy may be sufficient to cause a specific anti-tumor response and can prime the immune system as part of an adaptive immunotherapeutic strategy [28, 29]. There are several reports in the literature showing regression of malignancy after cryoablation [30-32]. Two specific cases at our center have highlighted this “abscopal” phenomenon, with one patient having regression of all biopsy proven metastatic disease after ablation of the primary tumor, and another patient experiencing complete necrosis of an 8-cm biopsy-proven RCC after ablation of a biopsy-proven metastatic lesion [33]. Cryoablation appears to have a unique mechanism in generating an intense inflammatory infiltrate by nature of its mechanism causing organelle and cell membrane rupture, and maintaining initial tumor vascularity. In the clinical setting of RCC, ablative monotherapy appears insufficient to create a clinically evident tumor-specific response in the majority of patients. However, it has been shown to cause suppression of tumor rechallenge in animals given combination anti-CTLA-4 therapy, with potent induction of antitumor immunity [34]. The availability of immune checkpoint blockade agents such as tremelimumab can thus unbridle the initial intense inflammatory responses generated by cryoablation, which may lead to greater immune responses and possible improved clinical benefit. Relevant to the current protocol, MD Anderson has a substantial experience with tissue ablation in both the metastatic and non-metastatic setting [35-37]. The majority of patients (>90%) who come in for soft tissue/bone cryo for pain palliation stay overnight and are discharged the next day. Those with very small lesions can be treated as outpatients, but these account for <10% of our population. Only one patient in the last year was hospitalized for 2 days.

Combination therapies: Because of the modest responses seen with checkpoint blockade monotherapy, various studies are starting to evaluate combining these with another immunologic primer, such as radiation therapy, antivascular therapy, hormonal therapy, and

with other forms of checkpoint blockade [38-40]. Combination therapy, particularly in the context of a potent immunologic primer such as tumor cryoablation, can lead to a significant "postvaccination" cascade [38].Toxicity in combination settings does not seem to be higher than monotherapy, but the combination of cryoablation and checkpoint blockade has not been tested in the human. Combination anti-CTLA-4 and ablation strategies are also being investigated for other cancers (http://clinicaltrials.gov/ct2/show/NCT01853618).

Current proposal: This pilot study will seek to support the hypothesis that the combination of cryoablation with tremelimumab is safe and well tolerated; secondarily we perform neoantigen evaluation and investigate measurable differences in the immune and clinical response generated by combination therapy as opposed to tremelimumab alone. We will recruit patients with mRCC who have a metastatic site (bone or soft tissue) amenable to cryoablation and either a primary site amenable to cytoreductive nephrectomy (current standard of care), a metastatic site amenable to metastasectomy or repeated biopsy. In effect, this study will allow for the first time the systematic study of the "abscopal" effect in the human, enabling an understanding of the mechanisms that lead to an effective antitumor response by checkpoint blockade when combined with a potent primer, leveraging the discovery of neoantigens expressed by the individual's tumor. One consequent therapeutic strategy resulting from these studies would be to combine tremelimumab with peptide vaccines as part of a separate protocol.

Dose Justification:
Tremelimumab has been administered to approximately 1,000 subjects as monotherapy at doses ranging from 10 mg/kg Q4W to 15 mg/kg Q12W. A Phase 1b/2 clinical study in melanoma (A3671002; Camacho et al, 2009) has shown comparable efficacy and overall AE rates at the 2 dosing levels, although more Grade 3/4 AEs were seen at a dose of 10 mg/kg Q4W (27% Q4W vs. 13% 15 mg/kg Q12W). The difference in Grade 3-4 AEs was largely due to differences in the incidence of ≥ Grade 3 diarrhea (21% vs. 9%). Nevertheless, all Grade 3 or 4 AEs and SAEs were manageable and reversible when appropriate intervention was applied. Subsequent large Phase 2 and 3 studies with Tremelimumab in melanoma used the regimen of 15 mg/kg Q12W. However, re assessment of both the safety and the efficacy conclusions regarding the dosing regimen comparisons in Study A3671002 is warranted. The incidence of diarrhea might be reduced with the implementation of current clinical practice guidelines for the management of immune-related toxicity (Kaehler et al, 2010; Weber, 2012; see Section 4.5.7). In addition, there was an imbalance between the treatment arms of the Phase 2 portion of Study A3671002. A greater proportion of subjects in the 10-mg/kg arm had baseline characteristics that were associated with poor prognosis (e.g., metastatic burden [M1c], lactate dehydrogenase [LDH] > upper limit of normal [ULN], Eastern Cooperative Oncology Group [ECOG] performance status > 0, and C-reactive protein [CRP] > 1.5 × ULN), which could have influenced the survival outcome in this small study. Cox-proportional hazard modeling, accounting for imbalances in prognostic factors, resulted in hazard ratios favoring the 10 mg/kg Q4W group, ranging from 0.7 to 0.8, although the differences were not significant ($p > 0.05$).

A retrospective exposure and survival analysis of 293 subjects treated with Tremelimumab in a Phase 3 study in melanoma showed better OS in subjects with higher exposure. The

median OS was 18.4 months for the high-AUC (≥ 123,665 µg.hr/mL) group compared to 9.0 months for the low-AUC (< 123,665 µg.hr/mL) group (HR 0.5; p < 0.001). Similar results were observed in a large Phase 2 study in patients with refractory or relapsed melanoma. The target trough concentration of Tremelimumab is estimated to be ~ 30 µg/mL based on enhanced IL-2 release (in vitro) and antitumor activity (in vivo) in preclinical studies. Pharmacokinetic simulations indicate that following Tremelimumab at a dose of 10 mg/kg Q4W, approximately 90% of subjects are expected to be above the target concentration of ~ 30 µg/mL compared to ~ 50% with 15 mg/kg Q12W. Refer to the current tremelimumab Investigator's Brochure for a complete summary of non-clinical and clinical information including safety, efficacy and pharmacokinetics

As of the data cutoff dates (1 November 2015 for monotherapy studies and 15 April 2015 to 12 July 2015 for combination therapy studies), 34 sponsored clinical studies have been conducted as part of the tremelimumab clinical development program. Of these, 13 studies have completed and 21 are ongoing. Eight tremelimumab monotherapy studies have been completed and 3 are ongoing. As of the data cutoff date of 1 November 2015, 973 patients received tremelimumab in completed monotherapy studies and the ongoing Study D4881C00024 and 569 patients have been treated in the ongoing blinded Phase IIb monotherapy Study D4880C00003 [DETERMINE]). In the 3rd ongoing monotherapy study (D4884C00001), no patients have been treated as of the data cutoff. In addition, approximately 59 patients have been treated with tremelimumab in monotherapy arms of combination studies. Five studies of tremelimumab in combination with other anticancer agents have been completed and 18 are ongoing. In total, 250 patients with a variety of tumor types have received tremelimumab in combination with other anticancer agents in these studies.

3.0 ELIGIBILITY CRITERIA

For entry into the study, the following criteria MUST be met. Any exceptions from the protocol-specific selection criteria must be approved by the Principal Investigator, and MD Anderson Cancer Center-IND Office, as the IND Sponsor and IRB, prior to enrollment.

3.1 Inclusion Criteria

1. Patients included in the study must be ≥ 18 years old.
2. Metastatic renal cell carcinoma (clear cell or non clear cell).
   a. One metastatic site amenable to cryoablation.
   b. Patients with a single metastatic site may be enrolled if that site is amenable to ablation and the patient is eligible for cytoreductive nephrectomy; however these patients will not be counted in secondary measures of response unless there is new disease detected during follow up.
   c. Eligible for cytoreductive nephrectomy, metastasectomy, or repeated biopsy. Biopsy site cannot be lung, mediastinal lymph node, or bone (unless soft tissue component).

3. Patients with any number of prior therapies with anti-angiogenic agents or immunotherapy with the exception of any previous anti-CTLA-4 directed agents are

allowed. A 2 week washout period is required for all agents, except for bevacizumab where a 4 week washout is required.

4. Performance status with ECOG score ≤ 2. Patients with performance status of 3 may be considered as long as the decline has been of short duration (< 1 month), and is due to their malignancy and not a comorbid condition (example: pain limiting activity).

5. Patient's with an International Metastatic renal cell carcinoma Database Consortium (IMDC or Heng) score of 3 or less will be included. Score greater than 4 will be excluded. 1 point each: requirement of systemic treatment for metastatic disease less than 1 year of original diagnosis of renal cell carcinoma, a serum calcium greater than 10, anemia, neutrophilia, thrombocytosis, ECOG performance status ≥ 2.

6. No history of autoimmune disorders.

7. Patients must have normal organ and marrow function as defined below:
   - WBC ≥ 2000/uL.
   - ANC ≥ 1000/uL.
   - Platelets ≥ 75 x 10^3/uL.
   - Hemoglobin ≥ 9 g/dL.
   - Creatinine ≤ 2.0 mg/dL.
   - ALT ≤ 3.0 x ULN for patients without liver metastases. For patients with liver metastasis ALT ≤ 5 x ULN is allowed.
   - Bilirubin ≤ 1.5 x ULN (except for patients with Gilbert's Syndrome, who must have a total bilirubin ≤ 3mg/dL).

8. Ability to understand and willingness to sign a written informed consent document.

9. Females of childbearing potential who are sexually active with a non-sterilized male partner and non-sterilized males must use a highly effective method of contraception for 28 days prior to the first dose of investigational product, and must agree to continue using such precautions for 180 days after the final dose of investigational product; cessation of contraception after this point should be discussed with a responsible physician. Periodic abstinence, the rhythm method, and the withdrawal method are not acceptable methods of contraception. They must also refrain from egg cell donation for 180 days after the final dose of investigational product;
   - Females of childbearing potential are defined as those who are not surgically sterile (ie, bilateral tubal ligation, bilateral oophorectomy, or complete hysterectomy) or postmenopausal (defined as 12 months with no menses without an alternative medical cause);

- A highly effective method of contraception is defined as one that results in a low failure rate (ie, less than 1% per year) when used consistently and correctly. The acceptable methods of contraception are: Barrier Method (e.g. male condom with spermicide, copper T intrauterine device, or levonorgesterl-releasing intrauterine system - Mirena®) or Hormonal Methods (e.g. implants, hormone shot or injection, combined pill, minipill, or patch).

3.2 Exclusion Criteria

1. Unresolved toxicities from prior anticancer therapy, defined as having not resolved to NCI CTCAE v4.03 Grade 0 or 1 with the exception of alopecia and laboratory values listed per the inclusion criteria.
2. Known or suspected autoimmune disease. Patients with a history of inflammatory bowel disease (including Crohn's disease and ulcerative colitis) and autoimmune disorders such as rheumatoid arthritis, systemic progressive sclerosis [scleroderma], Systemic Lupus Erythematosus or autoimmune vasculitis [e.g., Wegener's Granulomatosis] are excluded from this study. Patients with a history of Hashimoto's thyroiditis only requiring hormone replacement, Type I diabetes, or psoriasis not requiring systemic treatment, or conditions not expected to recur in the absence of an external trigger are allowed to participate.
3. Any condition requiring systemic treatment with corticosteroids (>10mg daily prednisone equivalents) or other immunosuppressive medications within 14 days prior to first dose of study drug. Inhaled steroids and adrenal replacement steroids doses >10mg daily prednisone equivalents are permitted in the absence of active autoimmune disease.
4. Any underlying medical or psychiatric condition, which in the opinion of the Investigator, will make the administration of study drug hazardous or obscure the interpretation of AEs, such as a condition associated with frequent diarrhea.
5. Patients with untreated brain metastases. Gamma knife or other stereotactic radiation, whole brain radiation, and surgical resection are considered adequate treatment; patients should be off steroids for 14 days prior to enrollment.
6. Major surgery within 4 weeks of enrollment.
7. History of other malignancies, other than non-melanoma skin cancer, Ta or T1 (low grade) bladder carcinomas, or other low grade cancer of very low clinical impact, unless in complete remission and off therapy for that disease for at least 2years.
8. Uncontrolled intercurrent illness including, but not limited to, ongoing or active infection, history of congestive heart failure, unstable angina pectoris, cardiac arrhythmia, or psychiatric illness/social situations that would limit compliance with study requirements.
9. Known HIV, Hepatitis B, or Hepatitis C.
10. Untreated symptomatic spinal cord compressions.
11. *Any* non-oncology live or attenuated vaccine therapy used for prevention of

infectious diseases within 30 days prior to the first dose of tremelimumab; if patients is enrolled, patient should not receive live vaccine during the study and 180 days after the last dose of tremelimumab.

12. Concomitant therapy with any of the following: IL-2, interferon or other non-study immunotherapy regimens; cytotoxic chemotherapy; immunosuppressive agents; other investigation therapies; or chronic use of systemic corticosteroids (used in the management of cancer or non-cancer-related illnesses).
13. Previous participation in tremelimumab or ipilimumab clinical trial or prior treatment with a CD137 agonist or CTLA-4 inhibitor or agonist.
14. Pregnant or Breastfeeding
15. Female subjects who are pregnant, breast-feeding or male or female patients of reproductive potential who are not employing an effective method of birth control from screening to 90 days after the last dose of tremelimumab monotherapy

4.0 DRUG INFORMATION (adapted from Investigator's Brochure)

4.1 Tremelimumab

Tremelimumab (formerly CP-675,206) is a human immunoglobulin G2 (IgG2) monoclonal antibody (mAb) being investigated as a cancer immunotherapeutic agent. Tremelimumab is specific for human cytotoxic T lymphocyte-associated antigen 4 (CTLA-4), with no crossreactivity to related human proteins.

4.1.1 *Mechanism of Action:*

Upon T cell activation, CTLA-4 expression acts to dampen immune responses by CTLA-4 relocation to the cell surface in order to modulate and eventually switch off T cell activation. Tremelimumab blocks the inhibitory effect of CTLA-4, and therefore enhances T cell activation.

4.1.2 *Pharmacology:*

Tremelimumab is administered by IV infusion. The absorption of tremelimumab upon extravascular administration has not been investigated. The mean VSS for tremelimumab is 81.2 mL/kg, which is typical of mAbs with limited distribution. No studies of human tissue distribution of tremelimumab have been conducted. The mean CL for tremelimumab is 0.132 mL/hr/kg. Similar to other mAbs without target-mediated drug disposition, tremelimumab is likely to be cleared from circulation byendothelial cell uptake and proteolysis. Since mAbs are not primarily cleared via hepatic/renal pathways, no impact of renal/hepatic functions is expected on tremelimumab elimination. No studies have been conducted in patients with renal/hepatic impairment.

4.1.3 *Pre-clinical Toxicology*

Complete information on the pre-clinical toxicology studies can be found in the tremelimumab Investigator Brochure (IB).

4.1.4 *Pharmacokinetics of Tremelimumab in Patients*

Shown in Table 1 below. Additional information available in Appendix 1.

Table 1. Mean (SD) Tremelimumab PK Parameters Following Single and Multiple (Administered Monthly) 10 mg/kg IV Doses

First dose	Dose (mg/kg)	N	$AUC_{0-τ}$ (µg·hr/mL)	C_{max} (µg/mL)	C_{trough} (µg/mL)	CL (mL/hr/kg)	Rac
	10	22	56,800 (13,200)	226 (70)	42.9 (14.1)	--	--
Steady state	10	11	74,800 (26,200)	287 (111)	52.2 (29.9)	0.148 (0.049)	1.26 (0.42)

AUC0-τ = area under the dosing interval concentration-time curve; CL = clearance; Cmax = maximum plasma concentration; Ctrough = plasma concentration at the end of the dosing interval; IV = intravenous; N = total number of patients; PK = pharmacokinetic; Rac = accumulation ratio based on AUC0-τ; SD = standard deviation. Note: Cmax was measured as the plasma concentration of tremelimumab 1 hour after the end of infusion of tremelimumab.

4.1.5 *Clinical Safety*

Tremelimumab has been administered as single-agent treatment to patients participating in 9 sponsored clinical studies, 1 of which continues to follow patients. In total, more than 1,300 patients with a variety of tumor types have been treated in these studies, including over 1,000 patients treated with tremelimumab. In addition, 116 patients with a variety of tumor types have received tremelimumab in combination with other anticancer agents in 5 clinical trials. In clinical subjects, tremelimumab exhibits a biphasic pharmacokinetic (PK) profile following IV infusion. The mean systemic exposure parameters Cmax (maximum plasma concentration) and AUC (area under the plasma concentration-time curve) increase with dose in an approximately proportional manner. The clearance (CL) of tremelimumab is 0.132 mL/h/kg, the volume of distribution at steady state (VSS) is 81.2 mL/kg, and the terminal phase half-life is 22.1 days. These values are consistent with those of natural IgG2.

As of the data cutoff date of 30Aug2013, 973 subjects have received tremelimumab monotherapy (not including subjects in the ongoing, blinded Phase 2 study, D4880C00003) whereas 116 subjects have received tremelimumab in combination with other agents. The majority of these subjects had melanoma and was treated at a dose of 15 mg/kg Q90D. The profile of AEs and the spectrum of event severity have remained stable across the tremelimumab clinical program and are consistent with the pharmacology of the target.Events reported in > 5% of subjects treated with tremelimumab monotherapy and assessed by the investigator as treatment related (listed in descending order of frequency) were diarrhea, rash, pruritus, fatigue, nausea, vomiting, decreased appetite, headache, abdominal pain, and colitis.

The safety profile of tremelimumab has been consistent across trials with:
a) the majority of adverse events being inflammatory in nature and consistent with the proposed mechanism of action of tremelimumab (immune-related adverse events, IRAEs),
b) the same types of such immune-mediated events in the GI tract, skin, liver and endocrine system being reported and

c) most of these events being manageable with immune suppressive therapies (see below).

Figure 1.

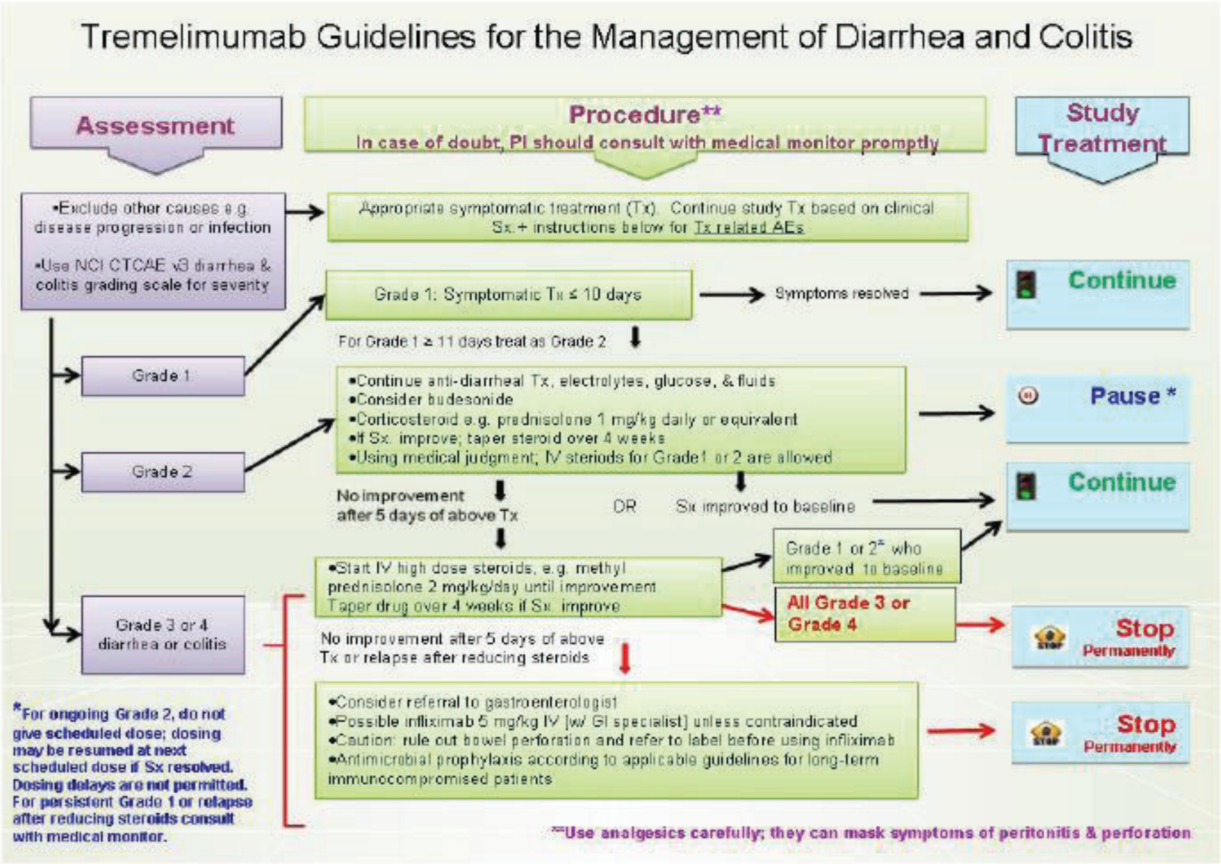

The most common IRAEs are colitis and diarrhea, rash, pruritus, deficiencies of endocrine organs (pituitary, adrenal or thyroid), hepatitis, or uveitis. Rare complications are bowel perforations (~1%) resulting from underlying severe colitis, which have required surgical intervention.

Drug-related Grade 3 or 4 serious AEs (SAEs) consist mostly of immune-related SAEs and include: rash/desquamation, pruritus, uveitis, speech impairment, abdominal pain, diarrhea/colitis, nausea/vomiting, transaminase elevation, adrenal insufficiency, panhypopituitarism and atrial fibrillation. Please refer to the most recent version of Investigator's Brochure (IB) for the latest update on SAEs.

4.1.5.1 *Immune-related events:*

The AEs seen with tremelimumab and ipilimumab are similar, and associated with activation of the immune system. Potential immune-mediated events observed with the use of tremelimumab include those of the skin (vitiligo and cutaneous leukocytoclastic vasculitis), thyroid gland (hypo- and hyperthyroidism including autoimmune thyroiditis),

pituitary gland [hypophysitis (presenting with adrenal insufficiency and/or diabetes insipidus), hypopituitarism], diarrhea/colitis, mucosal inflammation, lipase elevation, autoimmune hepatitis, interstitial nephritis, rheumatoid arthritis, uveitis, retinopathy, immune-mediated thrombocytopenia, sarcoidosis and Evans' syndrome. Many of these events present in the first cycle of treatment.

In the Tremelimumab single-agent studies, 490 patients reported 1,041 treatment-emergent SAEs in 588 cases, regardless of causality. In the combination studies, 46 patients reported 91 SAEs in 52 cases due to any cause. The following all-causality SAEs were reported most frequently (≥ 2% of the total number of events) across the program: diarrhea (109 cases; 9.6% of all SAEs), disease progression (89 cases; 7.9% of all SAEs), malignant melanoma (59 cases; 5.2% of all SAEs), colitis (48 cases; 4.2% of all SAEs), dehydration (37 cases; 3.3% of all SAEs), pyrexia (40 cases; 3.5% of all SAEs), vomiting (38 cases; 3.4% of all SAEs), and nausea (25 cases; 2.2% of all SAEs).

4.1.5.2 *Drug Related Deaths:*
As of November 15 2009, there had been 132 all-causality deaths among the approximately 1,000 tremelimumab-treated patients that met the reporting criteria for an SAE (120 in single-agent studies, 7 in combination studies, and 5 in investigator-led research studies). Of these, 98 (89 in single agent studies, 5 in combination studies, and 4 in investigator-led research studies) were ascribed to progression of the malignancy under study, 13 (10 in single-agent studies, 2 in combination studies, and 1 in an investigator-led research study) were due to SAEs judged to be related or possibly related to tremelimumab, and 21 (all in single-agent studies) were ascribed to other causes. The treatment-related deaths were attributed to colitis, colonic/intestinal perforation, diverticular perforation/diverticulitis, pulmonary embolism, pneumonia, septic shock, cardiac arrest, electrolyte imbalance, sudden death, and hemorrhage.

4.1.5.3 *Clinical Efficacy:*
Across the clinical development program for tremelimumab, and that of the related anti-CTLA-4 antibody ipilimumab, a pattern of efficacy has emerged that appears to be consistent across tumor types for this mechanism of action. Response rates to anti-CTLA-4 antibodies are generally low, approximately 10%. However, in patients who respond, the responses are generally durable, lasting several months even in patients with aggressive tumors such as refractory metastatic melanoma. The impact on PFS can be small, as some patients may have perceived progression of their disease in advance of developing disease stabilization or a tumor response. This early progression is thought to be due to the delay in activation of the immune system with anti-CTLA-4 antibodies, as these agents do not target the tumor directly. However, the proportion of patients with durable responses or stable disease can lead to a significant prolongation of overall survival (OS).

4.1.6 *Formulation*
Tremelimumab Drug Product is formulated at a nominal concentration of **20 mg/mL** in 20 mM histidine/histidine hydrochloride, 222 mM trehalose dihydrate, 0.02% (w/v) polysorbate 80, 0.27 mM disodium edetate dihydrate (EDTA), pH 5.5. The drug product

is a clear to opalescent, colorless to yellowish liquid. Drug product is supplied as a sterile IV solution, **packaged in 20 mL clear glass vials** with a rubber stopper and aluminum seal. Each vial contains 20 mg/mL of tremelimumab with a nominal fill of 20 mL (accounting to **400 mg/vial**). Vials containing tremelimumab must be stored in the refrigerator at 2-8°C and should not be frozen. Tremelimumab is dosed as an IV infusion with dilution in sterile, normal saline for injection. The product should be protected from light when not in use.

4.1.7 *Packaging and Labeling*

Drug product is supplied as a sterile IV solution, packaged in 20 mL clear glass vials with a rubber stopper and aluminum seal. Each vial contains 20 mg/mL of tremelimumab with a nominal fill of 20 mL (accounting to 400 mg/vial).

Medimmune will provide Tremelimumab at no cost for this trial.
Study medication will be provided as open-label containers. The labels will contain the batch number, content, storage conditions and dispensing instructions along with the Investigational New Drug (IND) caution statement.

4.1.8 *Storage, Handling and Dispensing of Tremelimumab*

4.1.8.1 *Storage*

Study treatments must be stored in a secure area under the appropriate physical conditions for the product. Access to and administration of the study treatment will be limited to the investigator and authorized site staff. Study mediation must be dispensed or administered only to subjects enrolled in the study and in accordance with the protocol. The study medication should be stored at refrigerated temperatures (2 to 8°C) and should not be frozen. Only sterile, normal saline for injection should be used as the diluent for tremelimumab IV injection, as required. The product should be protected from light when not in use.

4.1.8.2 *Handling and Disposal*

Study drug will be disposed of according to local institutional standard operating procedures.

4.1.8.3 *Dispensing*

It is the responsibility of the Investigator to ensure that tremelimumab is only dispensed to study subjects. The tremelimumab must be dispensed only from official study sites by authorized personnel according to local regulations.

4.1.9 *Drug Ordering and Accountability*

Initial Orders:

Drug acquisition, inventory control and dispensation will be managed by the investigational pharmacy.

Re-supply:

Re-supply orders will be submitted by investigational pharmacy based on available inventory and subject accrual rates.

Tremelimumab Accountability:
It is the responsibility of the Investigator to ensure that a current record of tremelimumab disposition is maintained at each study site where tremelimumab is inventoried and disposed. Records or logs must comply with applicable regulations and guidelines, and should include:

- Amount received and placed in storage area.
- Amount currently in storage area.
- Label ID number or batch number and use date or expiry date.
- Dates and initials of person responsible for each tremelimumab inventory entry/movement.
- Amount dispensed to and returned by each subject, including unique subject identifiers.
- Amount transferred to another area/site for dispensing or storage.
- Non-study disposition (e.g., lost, wasted, broken).
- Amount destroyed at study site.

Destruction of Tremelimumab:
Tremelimumab is to be destroyed on site, it is the Investigator’s responsibility to ensure that arrangements have been made for disposal and that procedures for proper disposal have been established according to applicable regulations, guidelines, and institutional procedures. Appropriate records of the disposal must be maintained.

4.1.10 *Preparation of Tremelimumab*
Tremelimumab is supplied as a sterile IV solution, filled in 20 mL clear glass vials with a rubber stopper and aluminum seal. Each vial contains 20 mg/mL (with a nominal fill of 20 mL accounting to 400 mg/vial) of tremelimumab, in an isotonic solution at pH 5.5. Vials containing tremelimumab must be stored in the refrigerator at 2-8°C. The 20 mg/mL solution will be diluted into a saline bag for IV infusion. Vials containing tremelimumab may be gently inverted for mixing, but should not be shaken.

For dose preparation steps, the following ancillary items are required:

- IV infusion bags of 0.9% sodium chloride injection (250 mL size). Saline bags must be latex-free and can be made of polyvinyl chloride (PVC) or polyolefins (eg, polyethylene), manufactured with bis (2-ethylhexyl) phthalate (DEHP) or DEHP-free.
- IV infusion lines made of PVC/DEHP or PVC/tri octyl trimellitate (TOTM) or polyethylene or polyurethane. All DEHP-containing or DEHP-free lines are acceptable. Lines should contain a 0.22 or 0.2 µm in-line filter. The in-line filter can be made of polyethersulfone (PES) or polyvinylidine fluoride DRF (PVDF). Lines containing cellulose-based filters <u>should not</u> be used with tremelimumab.
- Catheters/infusion sets made of polyurethane or fluoropolymer with silicone and stainless steel and/or PVC components.
- Syringes made of polypropylene and latex-free. Polycarbonate syringes <u>should not</u> be used with tremelimumab.

- Needles made of stainless steel.

Example: A subject weighing 85 kg will require 42.5 mL (3 vials) of investigational product. Remove 42.5 mL of saline from the commercial IV bag. Add the 42.5 mL of investigational product to the IV bag and gently mix by inverting up and down.

4.1.11 *Administration*
Tremelimumab is administered as an IV infusion only.

<u>Dose Calculations</u>
Subject weight at baseline should be used for dosing calculations unless there is a ≥ 10% change in weight.
The dose will be calculated using the following formula:

$$\text{Dose (mL)} = \frac{[\text{subject weight (kg)} \times \text{dose level (10 mg/kg)}]}{\text{drug concentration (20 mg/mL)}}$$

The corresponding volume of investigational product should be rounded to the nearest tenth of a mL (0.1 mL). Each vial contains a small amount of overage and the overage should be utilized as much as possible before using another vial.
The number of vials required for dose preparation is the next greatest whole number of vials from the following formula:

Number of vials = Dose (mL) ÷ 20 (mL/vial)

For example, a patient weighing 114 kg (250 lb) would be administered 1140 mg of tremelimumab (114 kg x 10 mg/kg = 1140 mg) with an infusion volume of 57 mL (1140 mg ÷ 20 mg/mL = 57 mL) at a rate of approximately 0.63 mL/min (57 mL ÷ 60 minutes) in 60 minutes.

5.0 TREATMENT PLAN

This is a randomized pilot trial of anti-CTLA-4 (Arm A) vs. cryoablation + anti-CTLA-4 (Arm B) for patients with advanced/metastatic renal cell carcinoma who are eligible for cytoreductive nephrectomy, metastasectomy, or repeated biopsy. Each patient in both Arms A and B will be given the anti-CTLA-4 monoclonal antibody tremelimumab at 10mg/kg every month for 2 doses prior to surgery, metastasectomy, or repeated biopsy. Cytoreductive nephrectomy, metastasectomy, or repeated biopsy will occur 4-6 weeks after the 2nd dose of tremelimumab. After 4 weeks post-surgery or biopsy, patients will continue to receive anti-CTLA-4 every 4 weeks for 3 doses and every 12 weeks after that until progression. Patients in Arm B will additionally receive cryoablation of a metastasis 2-6 days prior to the first dose of tremelimumab. Notice: Treatment schedules shall have a standing window of allowance of +/- 2 days when dosed every 4 weeks and +/- 5 days when dosed every 12 weeks.) Any treatment day that falls on a weekend or holiday will be scheduled on the next business day.

For treatment or dose modification questions, please contact Padmanee Sharma MD, PhD. Matthew Campbell MD or Surena F. Matin MD. No investigational or commercial agents or therapies other than those described below may be administered with the intent to treat the patient's malignancy.

5.1 AGENTS AND PROCEDURES

5.1.1 Cryoablation

Patients randomized to cryoablation will undergo cryoablation by one of the collaborating Interventional Radiologists (Dr Alda Tam, Kamran Ahrar, Rahul Sheth or other designees in case both are absent) per current IR procedures, as follows:

An appropriate metastatic site, determined by discussion between urology or medical oncology and IR physicians, will undergo cryoablation under sedation or general anesthesia as per current IR protocols.

5.1.2 Agent Administration

All patients will receive a course of 2 pre-intervention doses of tremelimumab; those undergoing cryoablation can receive the first dose 2-6 days after cryoablation. Treatment will be administered as follows:

Tremelimumab will be administered intravenously at a dose of 10mg/kg as an IV solution of 10 mg/kg at a rate of 250 mL/hr, followed by observation. Patients will receive the first dose at Week 1, second dose at Week 5. After surgery or biopsy subjects will receive one dose of investigational product Q4W for 3 doses and every 12 weeks after that until progression.

5.1.3 Cytoreductive nephrectomy, metastasectomy, or repeated biopsy

All patients will have their primary kidney tumor, metastasectomy, or repeated biopsy removed 4-6 weeks after the 2nd dose of tremelimumab.

Surgery can be performed by any means deemed appropriate by the treating surgeon (open, laparoscopic, robotic) and according to good medical practice. A partial nephrectomy is allowed if that procedure is indicated in the patient's best interest.

Biopsy locations must allow the use of at least an 18 gauge needle to perform a core biopsy as deemed by an interventional radiologist. Bone biopsies are allowed if a soft tissue component of the tumor exists. Should a patient have the complete disappearance of the reference lesion used as the baseline biopsy, but a different lesion persists or appears after treatment with tremelimumab, the remaining lesion may replace the original lesion for the on treatment biopsy.

5.2 Duration of Therapy and Criteria for Treatment Delay

Dosing delays are not permitted (except per protocol specified window of +/- 2 days when dosed every 4 weeks and +/- 5 days when doses every 12 weeks).Toxicity management guidelines for anti-CTLA-4 monoclonal antibodies have been developed and published in the past 5 years (Kaehler and Hauschild, 2011; Weber et al, 2012). Specific detailed management guidelines have been created for diarrhea and/or colitis-related events (Figure1). However, for management of most other toxicities follow the guideline in Table 2.

Table 2 General Toxicity Management Guideline (CTCAE Version 4.03)

Condition	Management
Onset of any toxicity	• Rule out alternative etiology<br>•
CTCAE Grade 1	• Provide symptomatic treatment.<br>• Possible topical steroids if applicable.<br>• If symptoms resolve, continue tremelimumab dosing.<br>• If symptoms persist >5 days, treat as CTCAE Grade 2.
CTCAE Grade 2	• Provide symptomatic treatment<br>• Do not give scheduled dose with the exception of grade 2 skin toxicity, grade 2 amylase or lipase elevation or asymptomatic grade 2 endocrinopathy that are asymptomatic and controlled with hormone replacement therapy; dosing may be resumed at next scheduled dose if symptoms are resolved.<br>• Dosing delays are not permitted (except per protocol specified window of +/- 2 days when dosed every 4 weeks and +/- 5 days when dosed every 12 weeks.).<br>• Consider oral or intravenous (IV) steroids at the onset of symptoms. Taper steroid over 4 weeks if symptoms improve<br>• For persistent Grade 2 start high dose IV steroids
CTCAE Grade 3	• Start high dose IV steroids at the onset of the symptoms with the exception of asymptomatic amylase or lipase abnormalities that are not associated with symptoms or clinical manifestations of pancreatitis do not require a dose delay. It is recommended to consult with the PI for Grade 3 amylase or lipase abnormalities.<br>• Provide symptomatic treatment<br>• Permanent discontinuation* of tremelimumab for CTCAE Grade 3 events thought to be drug-related. Exception are endocrinopathies that are asymptomatic and controlled with hormone replacement therapy, and rash or other skin disorders
CTCAE Grade 4	• Start high dose IV steroids at the onset of the symptoms<br>• Provide symptomatic treatment<br>• Permanent discontinuation* of tremelimumab for all Grade 4 events
Steroid refractory toxicity (no improvement after 5 days on high dose IV steroids) or relapse after reducing high dose steroids	• Continue symptomatic treatment and steroids<br>• Possible infliximab 5 mg/kg IV for gastro-intestinal toxicities unless contraindicated [consult with GI specialist]. Caution: rule out bowel perforation and refer to label before using infliximab.<br>• For patients with increased AST/ ALT, or Total Bilirubin levels, consider Mycophenolate

*Patients will not receive any subsequent dose, but will remain on study and follow the other procedures required from the study (e.g. follow up procedures, imaging follow-up, blood sample collections). In addition, sites will be encouraged to provide scans done after disease progression for other therapies in subjects who permanently discontinue investigational product.

Guidelines for Omitting (Skip) the next scheduled dose for drug-related adverse events

The following guidance (see Table 3 below) is for consideration when determining when to skip/hold the next scheduled dose of tremelimumab during management of drug-related toxicities.

When a scheduled dose of tremelimumab is skipped; the dosing may be resumed at the next scheduled dose if the symptoms have resolved to CTCAE Grade ≤ 1. Dose delays are not allowed (except per protocol specified window of +/- 2 days when dosed every 4 weeks and +/- 5 days when dosed every 12 weeks). No dose reductions are permitted for tremelimumab.

Table 3 Guideline for skipping a tremelimumab dose

Action	Condition
Consult with study PI and manager as a NCI CTCAE Grade 2.	For persistent related CTCAE Grade 1 AE (>10 days)

Table 3 Guideline for skipping a tremelimumab dose

Action	Condition
**Skip** scheduled tremelimumab dose	Subjects with AST/ALT 3× ULN or total bilirubin 2 × ULN or >3x ULN if history of Gilbert's Syndrome.<br>Subjects with any NCI CTCAE Grade 2 treatment-related laboratory abnormalities .<br>For ongoing NCI CTCAE Grade 2 related toxicities. Dosing may be resumed at next scheduled dose if the event is resolving and at Grade ≤ 1.<br>Exceptions to this are:<br>• Investigational product may be dosed for NCI CTCAE Grade ≤ 3 endocrine disorders, if asymptomatic and controlled with hormone replacement therapy. Skip investigational product dose for Grade ≥ 2 endocrinopathies that are under treatment and remain symptomatic.<br>• Grade 3 asymptomatic amylase or lipase elevation<br>• Grade 2 skin toxicity<br>• For hypersensitivity reactions and infusion reactions NCI CTCAE Grade ≤ 2: slow the infusion rate or temporally pause the infusion, medicate the subject with symptomatic therapies (ie, anti-histaminic drugs), and consider pre-medication per institutional guidelines. Consult with the medical monitor as needed.<br>• Investigational product may be dosed for NCI CTCAE Grade 2 rash or other skin disorders; skip dose for Grade 3 skin disorders, except investigational product may be dosed for vitiligo any grade.

ALT = alanine transaminase; AST = aspartate transaminase; NCI CTCAE = National Cancer Institute Common Terminology Criteria for Adverse Events; ULN = upper limit of normal.

During the study, patients may require immunosuppressive medications such as steroids for management of underlying disease, treatment-related toxicity, or unrelated conditions. If symptoms resolved to CTCAE Grade ≤1, tremelimumab dosing may be resumed during steroid taper. Patients with adrenal insufficiency may take daily prednisone or equivalent therapy for their endocrinopathy while receiving tremelimumab treatment. Topical and inhaled steroids in standard doses are allowed.

Guidelines for Permanent dosing discontinuation [Safety related]

Table 4 below lists the safety related conditions when patients must be permanently

discontinued from tremelimumab treatment. Additionally, tremelimumab should be permanently discontinued for any adverse event, which in the opinion of the investigator or sponsor, contraindicate further dosing. When tremelimumab dosing is permanently discontinued, continue to follow the patient (e.g. they will follow the other procedures required from the study; follow up procedures, imaging follow-up, blood samples collection etc.).

Table 4 Guideline for Permanent Discontinuation of tremelimumab

Action	Condition
**Permanent Discontinuation of tremelimumab**	CTCAE Grade ≥3 related diarrhoea or colitis<br>AST or ALT > 8 x ULN or total bilirubin > 3x ULN or >5x ULN if history of Gilbert's syndrome<br>ALT > 3x ULN with total bilirubin > 2 x ULN<br>CTCAE Grade ≥3 hypersensitivity reaction or infusion reaction; Recurrent/persistent CTCAE Grade 2 hypersensitivity<br>CTCAE Grade ≥ 3 related endocrine disorders, if symptomatic and not controlled with hormone replacement therapy. [Tremelimumab may de dosed for CTCAE Grade ≤ 3 endocrine disorders, if asymptomatic and controlled with hormone replacement therapy]<br>CTCAE Grade 4 rash or other skin disorders [with the exception of vitiligo, which may be dosed regardless of severity]<br>Any CTCAE Grade 3 irAE<br>Any other CTCAE Grade 3 events thought to be drug-related if the following occurs with the exception of asymptomatic elevation of pancreas enzymes<br>• For AEs that DOES NOT downgrade to ≤ Grade 2 within 7 days (and to Gr1 or baseline at time of next scheduled dose) or resolve to ≤ Grade 1 or baseline within 14 days,<br>Any CTCAE Grade 3 laboratory abnormalities thought to be drug-related(non irAE) if the following occurs:<br>• For AEs that DOES NOT downgrade to ≤ Grade 2 within 7 days (and to Gr1 or baseline at time of next scheduled dose) or resolve to ≤ Grade 1 or baseline within 14 days,<br>Any CTCAE Grade 4 event thought to be drug related<br>Any patient who receives infliximab or any other TNF alfa inhibitor<br>If 2 consecutive doses are missed due to on-going related toxicities<br>Begins new investigational therapy, chemotherapy, cytokine therapy, or immunotherapy (including vaccines) must withdraw from treatment<br>Patient becomes pregnant

5.3.2 *Exceptions to Permanent Discontinuation of tremelimumab dosing*:

5.3.2.1 Endocrinopathies where clinical symptoms are controlled with appropriate hormone replacement therapy and, in the Investigator's opinion, continuing study drug administration is justified. **Note:** tremelimumab may not be restarted while the patient is being treated with systemic corticosteroids except for patients on stable doses of hormone replacement therapy such as hydrocortisone.

5.3.2.2 The following grade 4 hematologic toxicities in the absence of any clinical deterioration, bleeding, or infection:

- CD4 count (<50/mm^3)
- Hemoglobin (<6.5g/dL)
- Leukocytes (<1000/mm^3)
- Lymphopenia (<200/ mm^3)
- Neutropenia (<500/ mm^3)

5.3.2.3 Potentially reversible inflammation (< Grade 4), attributable to a local anti-tumor reaction and a potential therapeutic response. This includes inflammatory reactions at sites of tumor resections or in draining lymph nodes, or at sites suspicious for, but not diagnostic of metastasis;

5.3.2.4 Hospitalization for ≤ Grade 2 adverse events where the primary reason for hospitalization is to expedite the clinical work-up;

5.3.2.5 Patients with the following conditions where in the Investigator's opinion continuing study drug administration is justified:

- Ocular toxicity that has responded to topical therapy;
- Endocrinopathies where clinical symptoms are controlled with appropriate hormone replacement therapy.
- Asymptomatic elevation of amylase and lipase (pancreas enzymes)

Note: Tremelimumab may not be restarted while the patient is being treated with systemic corticosteroids except for patients on stable doses of hormone replacement therapy such as hydrocortisone.

5.4 Criteria for Removal from Study

Subjects MUST be discontinued from study therapy AND withdrawn from the study for the following reasons:

5.4.1. Disease progression (see section 7.2 for definition of disease progression)

5.4.2. The patient withdraws consent (subject's decision to withdraw for any reason).

5.4.3. Imprisonment or the compulsory detention for treatment of either a psychiatric or physical (e.g., infectious disease) illness.

5.4.4 At the discretion of the treating physician based on clinical benefit.

5.5 Immune Related Adverse Events (IRAEs): Definition, Monitoring and Treatment

Blocking CTLA-4 function may permit the emergence of auto-reactive T cells and resultant clinical autoimmunity. Rash/vitiligo, diarrhea/colitis, uveitis/episcleritis, hepatitis and hypopituitarism were drug-related, presumptive autoimmune events, now termed IRAEs, noted in previous ipilimumab and tremelimumab studies.

For the purposes of this study, an IRAE is defined as an AE of unknown etiology, associated with drug exposure and is consistent with an immune phenomenon[40]. Efforts should be made to rule out neoplastic, infectious, metabolic, toxin or other etiologic causes prior to labeling an AE an IRAE. Serological, immunological, and histological (biopsy) data should be used to support the diagnosis of an immune-mediated toxicity. Suspected IRAEs must be documented on an AE or SAE form.

Patients should be informed of and carefully monitored for evidence of clinically significant systemic IRAE (e.g., systemic lupus erythematosus-like diseases) or organ-specific IRAE (e.g., rash, colitis, uveitis, hepatitis or thyroid disease). If an IRAE is noted, appropriate work-up (including biopsy if possible) should be performed, and steroid therapy may be considered if clinically necessary (see Appendix E for suggested work-up and treatment of IRAEs).

It is unknown if systemic corticosteroid therapy has an attenuating effect on tremelimumab activity. However, clinical anti-tumor responses have been maintained in patients treated with corticosteroids and discontinued from tremelimumab. If utilized, corticosteroid therapy should be individualized for each patient. Prior experience suggests that colitis manifested as ≥ Grade 3 diarrhea requires corticosteroid treatment. See Tables 3-5 for additional details.

5.6 Infusion Reactions and Fever Associated with Tremelimumab

5.6.1 *Infusion Reactions*

Since tremelimumab contains only human protein sequences, it is less likely that any allergic reaction will be seen in patients. However, it is possible that infusion of tremelimumab will induce a cytokine release syndrome that could be evidenced by fever, chills, rigors, rash, pruritus, hypo- or hypertension, bronchospasm or other symptoms. No prophylactic pre-medication will be given unless indicated by previous experience in an individual patient.

The following treatment guidelines are suggested:

5.6.1.1 For <u>MILD SYMPTOMS</u> (e.g., localized cutaneous reactions such as mild pruritus, flushing, rash):

- Decrease the rate of infusion until recovery from symptoms, remain at bedside and monitor patient;
- Complete the tremelimumab infusion at the initial planned rate;
- Diphenhydramine 50 mg IV may be administered at the discretion of the treating physician and patients may receive additional doses with close monitoring;

- Premedication with diphenhydramine may be given at the discretion of the Investigator for subsequent doses of tremelimumab.

5.6.1.2 For MODERATE SYMPTOMS (any symptom not listed above [mild symptoms] or below [severe symptoms] such as generalized pruritus, flushing, rash, dyspnea, hypotension with systolic BP >80 mmHg):

- Interrupt tremelimumab;
- Administer diphenhydramine 50 mg IV;
- Monitor patient closely until resolution of symptoms;
- Corticosteroids may abrogate any beneficial immunologic effect, but may be administered at the discretion of the treating physician;
- Resume tremelimumab infusion after recovery of symptoms;
- At the discretion of the treating physician, tremelimumab infusion may be resumed at *one half the initial infusion rate, then increased incrementally to the initial infusion rate.*
- If symptoms develop after resumption of the infusion, the infusion should be discontinued and no additional tremelimumab should be administered that day;
- The next dose of tremelimumab will be administered at its next scheduled time and may be given with pre-medication (diphenhydramine and acetaminophen) and careful monitoring, following the same treatment guidelines outlined above;
- At the discretion of the treating physician additional oral or IV antihistamine may be administered prior to dosing with tremelimumab.

5.6.1.3 For SEVERE SYMPTOMS (e.g., any reaction such as bronchospasm, generalized urticaria, systolic blood pressure <80 mm Hg, or angioedema):

- Immediately discontinue infusion of tremelimumab, and disconnect infusion tubing from the subject;
- Consider bronchodilators, epinephrine 1 mg IV or subcutaneously, and/or diphenhydramine 50 mg IV, with solumedrol 100 mg IV, as needed.
- Patients should be monitored until the Investigator is comfortable that the symptoms will not recur;
- No further tremelimumab will be administered;

5.6.1.4 In case of late-occurring hypersensitivity symptoms (e.g., appearance within one week after treatment of a localized or generalized pruritus), symptomatic treatment may be given (e.g., oral antihistamine, or corticosteroids).

5.6.2 *Treatment of Tremelimumab Related Isolated Drug Fever*

In the event of isolated drug fever, the Investigator must use clinical judgment to

determine if the fever is related to the tremeilimumab or to an infectious etiology.

If a patient experiences isolated drug fever, for the next dose, pre-treatment with acetaminophen or non-steroidal anti-inflammatory agent (Investigator discretion) should be instituted and a repeated antipyretic dose at 6 and 12 hours after tremelimumab infusion should be administered. The infusion rate will remain unchanged for future doses.

If a patient experiences recurrent isolated drug fever following pre-medication and post dosing with an appropriate antipyretic, the infusion rate for subsequent dosing should be decreased to 50% of the previous rate. If fever recurs following infusion rate change, the Investigator should assess the patient's level of discomfort with the event and use clinical judgment to determine if the patient should receive further tremelimumab.

5.7 Prohibited and Restricted Therapies During the Study

5.7.1 *Prohibited Therapies*

The following restrictions are to be followed during the study:

- Live attenuated Vaccinations should be avoided during the study, for 28 days prior to tremelimumab treatment, and for at least 180 days after the last dose of tremelimumab.
- Inactivated vaccination during the study should be avoided +/- 28 days around any dose of tremelimumab
- Drugs with laxative properties and herbal or natural remedies for constipation should be avoided during the study and through 90 days post last dose because of potential exacerbation of diarrhea.
- Patients who begin new investigational therapy, chemotherapy, cytokine therapy, or immunotherapy (including vaccines) must not receive further treatment in this study.
- Immunosuppressive medications including, but not limited to systemic corticosteroids at doses not exceeding 10 mg/day of prednisone or equivalent, methotrexate, azathioprine, and tumor necrosis factor alpha (TNF-α) blockers.

Note:

- A. Use of immunosuppressive medications for the management of investigational product-related AEs or in subjects with contrast allergies is acceptable.
- B. Use of inhaled and intranasal corticosteroids is permitted
- C. Patients who receive even a single dose of infliximab or any other TNF alfa inhibitor must be permanently withdrawn from the study.

- For patients in renal cell carcinoma studies: Patients should not receive sunitunib within 3 months of a dose of tremelimumab, as acute renal failure has been reported with combination therapy of tremelimumab and sunitunib. It is not known whether there

could be a safety issue with combination of tremelimumab and other tyrosine kinase inhibitors.

Concomitant systemic or local **anti-cancer** medications or treatments are prohibited in this study while receiving tremelimumab treatments, other than palliative (pain controlling) radiation therapy (RT) in situations that are not clearly indicative for PD.

Patients may not use any of the following therapies during the study:

- Any non-study anti-cancer agent (investigational or non-investigational);
- Any other investigational agents;
- Any other (non-CA184024 related) CTLA-4 inhibitors or agonists;
- CD137 agonists;
- Immunosuppressive agents;
- Chronic systemic corticosteroids;
- Any non-oncology vaccine therapies used for the prevention of infectious diseases (for up to 30 days prior to or after any dose of study drug).

6.0 PATIENT EVALUATION

6.1 Screening Evaluation

Pre-study tests/evaluations that must occur within a defined time frame shall have a standing window of allowance that is equal to +/- 3 days. Sections 6.1 – 6.4 are summarized in Appendix D- Study Calendar.

6.1.1

Within 35 days of starting Study drug

- Height, weight, PS (ECOG), Vital Signs (BP, pulse, temperature)
- Imaging studies:
  - Contrast CT scans of the abdomen (preferred) or MRI of abdomen
  - Pelvic CT or MRI is optional, based on evidence or suspicion of disease
  - Chest X-ray (if lung metastases are evident on chest x-ray, CT of the chest should be obtained as well)
  - Bone scan
- Complete medical history and physical examination
- Electrocardiogram
- T-spot testing

6.1.2

Within 14 days of starting study drug

- Weight, PS (ECOG), Vital Signs (BP, pulse, temperature)
- List of concurrent medications
- Baseline Toxicity evaluation
- Laboratory testing:
  - CBC with differential/platelets
  - Serum chemistry must include: Creatinine, BUN, sodium, chloride, CO_2,

potassium, calcium, magnesium, phosphorus, glucose, albumin, total protein, total bilirubin, SGOT [AST], SGPT [ALT], alkaline phosphatase, bone alkaline phosphatase (ALP) and LDH.
- Autoimmune Panel: ESR, RF (Rheumatoid Factor), ANA (Antinuclear antibody) titer and pattern.
- ACTH, cortisol, TSH, free T4
- Amylase and lipase
- Pregnancy Test (Urine and/or Blood)
- SPEP (serum protein electrophoresis, for total immunoglobulin levels), and anti-TPO (thyroid peroxidase) titer

6.2 On-Study Evaluation

Notice: On-study tests/visits that must occur within a defined time frame not otherwise specified shall have a standing window of allowance that is equal to +/- 3 days.

6.2.1 **<u>Cryoablation (Arm B only)</u>**

Within 2-6 days of starting study drug. For delays that last longer than 3 weeks subject will be removed from study.

6.2.2 *Prior to each dose of tremelimumab, within 2 weeks prior to surgery or repeated biopsy, within 4 weeks after surgery or biopsy, and every 4 weeks thereafter for up to 3 months after surgery or biopsy*

- Physical examination, interim history pertaining to any change from baseline, current medications and treatment-related toxicities. Adverse events will be monitored using the NCI CTCAE Version 4.03.
- weight, Vital Signs (BP, pulse, temperature)
- Laboratory testing:
  - Baseline screening laboratory tests will not be repeated on week 1 for patients on Arm A.
  - CBC with differential/platelets
  - Serum chemistry must include: creatinine, BUN, sodium, chloride, bicarbonate potassium, calcium, magnesium, phosphorus, glucose, albumin, total protein, total bilirubin, SGOT [AST], SGPT [ALT], alkaline phosphatase, bone alkaline phosphatase (ALP) and LDH.
  - Serum chemistry: creatinine, BUN, sodium, chloride, bicarbonate potassium, calcium, magnesium, phosphorus, glucose, albumin, total protein, total bilirubin, SGOT [AST], SGPT [ALT], alkaline phosphatase, bone alkaline phosphatase (ALP) and LDH will be evaluated every 2 weeks for the first 2 months during tremelimumab infusions.
  - ACTH, cortisol, TSH, free T4
  - Amylase, lipase
  - SPEP and anti-TPO titer

6.2.3 Within 2 weeks prior to surgery or biopsy and within 4 weeks after surgery or biopsy

- Imaging studies: Re-staging scans or other disease assessment diagnostics during the study are at the discretion of the study collaborator or the principal investigator and shall be performed if disease progression is suspected:

  - Contrast CT scan of the abdomen (preferred) or MRI of abdomen
  - Pelvic CT or MRI based on suspicion of disease
  - Chest X-ray (if lung metastases are evident on chest x-ray, CT of the chest should be obtained as well)
  - Bone scan (only if clinically indicated)

6.2.4 Day of surgery will occur within 8-10 weeks from the start of study drug.
  - Nephrectomy
  - Metastasectomy
  - Biopsy
  - Tumor tissue collection

6.2.5 *Every 12 Weeks, beginning 3 months after surgery or biopsy:*
- Physical examination, interim history pertaining to any change from baseline, current medications and treatment-related toxicities. Adverse events will be monitored using the NCI CTCAE Version 4.03.

- Laboratory testing:
  - CBC with differential/platelets
  - Serum chemistry must include: creatinine, BUN, sodium, chloride, CO2, potassium, calcium, magnesium, phosphorus, glucose, albumin, total protein, total bilirubin, SGOT [AST], SGPT [ALT], alkaline phosphatase, and LDH. Bone alkaline phosphatase (ALP).
  - ACTH, cortisol, TSH, free T4
  - Amylase, lipase

- Imaging studies: Re-staging scans or other disease assessment diagnostics during the study are at the discretion of the study collaborator or the principal investigator and shall be performed if disease progression is suspected:

  - Contrast CT scan of the abdomen (preferred) or MRI of abdomen
  - Pelvic CT or MRI based on suspicion of disease
  - Chest X-ray (if lung metastases are evident on chest x-ray, CT of the chest should be obtained as well)
  - Bone scan (only if clinically indicated)

6.2.6 *Any response will be confirmed 4weeks after initial imaging showing a response.*

6.3 Post-Therapy evaluation

About 30, 60 and 90 days (window +/- 7 days) after the patient is taken off study drug, the following will be obtained and recorded:

- Physical examination, interim history pertaining to any change from baseline,

current medications and treatment-related toxicities.

- Laboratory testing:
  - CBC with differential/platelets
  - Serum chemistry must include: creatinine, BUN, sodium, chloride, bicarbonate potassium, calcium, magnesium, phosphorus, glucose, albumin, total protein, total bilirubin, SGOT [AST], SGPT [ALT], alkaline phosphatase and LDH.
  - ACTH, cortisol, TSH, free T4(30 Days post-therapy only)
  - Amylase, lipase(30 Days post-therapy only)SPEP, anti-TPO

- Imaging studies will be completed as clinically indicated as described below:
  - Contrast CT scan of the abdomen (preferred) or MRI of abdomen
  - Pelvic CT or MRI based on suspicion of disease
  - Chest X-ray (if lung metastases are evident on chest x-ray, CT of the chest should be obtained as well)
  - Bone scan (only if clinically indicated)

6.4 Survival Updates

Survival updates will take place at 6-month intervals (+/- 1 month) from the date of progression. These will be conducted by phone, e-mail or medical record review. No clinic visits or additional diagnostic studies are required by the protocol.

6.5 Safety, Tumor, Immunologic, and Molecular Evaluations

6.5.1 Safety Evaluation

All patients who receive at least one dose of tremelimumab will be considered evaluable for safety parameters. Additionally, any occurrence of a SAE from time of consent forward, up to and including follow-up visits will be reported. Refer to safety reporting section 10.

Safety will be evaluated for all treated patients using the NCI CTCAE version 4.03 (http://ctep.cancer.gov). Safety assessments will be based on medical review of AE reports and the results of vital sign measurements, physical examinations and clinical laboratory tests.

6.5.2 Tumor Evaluation

A challenge to the clinical development of tremelimumab is when and how to assess patients for antitumor effects. In many cases, antitumor effects are delayed or may only appear after a period of apparent disease progression. This could be attributed to the time required for activated T cells to infiltrate the tumor, which may result in an initial increase in tumor volume followed by tumor shrinkage. Based on the Response Evaluation Criteria in Solid Tumors (RECIST) and World Health Organization (WHO) criteria, a subgroup of patients who actually benefited from CTLA-4 inhibition may have been classified incorrectly as “non-responders.” Specific immune-related Response Criteria (irRC) have been utilized to prevent premature discontinuation of immune

therapies in solid malignancies.[43] However, for this trial RECIST 1.1 will be used in conjunction with the collaborating diagnostic radiologist to determine outcome and measurement.

A subject may stay on study if it is the in their best interest at the time of RECIST progression. A confirmatory tumor assessment may be performed no less than 4 weeks after the first indication of progression to provide additional data for the investigator to decide if the subject should stay in the trial or be discontinued due to clinical progression based on the investigator's decision.

6.5.2.1 Radiological Evaluation of Soft Tissue Lesions
CT/MRI imaging of the chest, abdomen and radionucleotide bone scan is required prior to initiating treatment and CT/MRI imaging of the chest, abdomen at each tumor assessment visit. Bone scan and CT/MRI of the pelvis are optional during the tumor assessment visits and may be done if clinically indicated. Similar methods of tumor assessment and similar techniques should be used to characterize each identified and reported lesion at baseline and during the treatment phase of the trial.

Measurable disease in viscera (liver or lung) is defined as per PCWG2 modified RECIST 1.1 as a lesion ≥10 mm in its longest diameter as measured with conventional techniques (ie, CT or MRI). For a lymph node to be considered measurable, the minimum diameter must be ≥20 mm in long axis when assessed by CT scan. All other lesions (or sites of disease) will be considered nonmeasurable disease.

All measurable lesions (up to a maximum of 2 lesions per organ and 5 lesions in total) will be identified as target lesions to be measured and recorded at baseline. The target lesions should be representative of all involved organs. Target lesions will be selected on the basis of size (ie, the largest area) and suitability for accurate, repeated measurements (either by imaging techniques or clinically). A sum of the diameters (long axis) for all target lesions will be calculated and reported as the baseline sum diameters. The baseline sum of diameters will be used as reference by which to characterize the objective tumor response.

Radionucleotide bone scan (if clinically indicated) outcome should be recorded as either new lesions or no new lesions. On bone scan, progression of bone metastases is defined as the appearance of 2 or more new bone lesions compared to the baseline scan. In the case of the first reassessment scan only, a confirmatory scan performed ≥4 weeks later needs to shows 2 or more additional new lesions (for a total of at least 4 new lesions seen since baseline) for progression to be documented (the date of progression is always the date of the first scan showing the change). For scans after the first reassessment, a confirmatory scan performed ≥4 weeks later needs to show the persistence of or an increase in the number of bone lesions compared to the prior scan.

CR – complete remission	Disappearance of all target lesions
PR – partial remission	30% decrease in the sum of the longest diameter of target lesions

PD – progressive disease	20% increase in the sum of the longest diameter of target lesions
SD – stable disease	Small changes that do not meet the above criteria

The PI, treating physician,and radiology collaborator will complete tumor measurements will complete tumor measurements and document in the medical record.

6.5.2.2 Treatment Beyond Progression
The appearance of a new lesion does not by itself satisfy the criteria for confirmed progressive disease. Rather, the tumor burden imposed by the new lesions must be evaluated within the context of the total tumor burden (ie, preexisting plus new lesions). Confirming progression in measurable lesions requires 2 assessment time points. The first must occur 12 weeks after the last dose of tremelimumab is administered (unless otherwise clinically indicated) and the second occurring ≥6 weeks after the first. Progression declared at the first time point remains unconfirmed unless assessments at the second time point demonstrate continuing or worsening progression. In some circumstances, it may be difficult to distinguish residual disease from normal tissue. When the evaluation of complete response depends on this determination, it is recommended that the residual lesion be investigated (eg, fine needle aspirate or biopsy) before confirming the complete response status.

6.5.3 Immunologic Evaluation

Tumor tissue and blood samples will be collected on laboratory protocol PA13-0291 for immune monitoring as previously published,[45-48] under the supervision of the Immunotherapy Platform. In tumor tissues, immunohistochemical studies will be performed to evaluate CD4 and CD8 T cells. In peripheral blood, we will also evaluate T cell populations including CD4 and CD8 cells in pre and post therapy samples.

Up to one hundred (150) ml of blood will be drawn at the visits outlined in Section 10.0. These samples will be labeled with confidential identification numbers and provided to Immunotherapy platform for immunological assessments. A detailed laboratory manual highlighting the blood collection and processing procedures will be prepared.

All blood collection will be compliant with institutional safety standards and will not exceed the maximum blood draw per venipuncture policy.

Tissue Collection: Tumor tissue will be collected for immune tests as described above.

7.0 Statistical Considerations

7.1 Preliminaries and Objectives

This is a randomized pilot trial of anti-CTLA-4 (Arm A) vs. cryoablation + anti-CTLA-4 (Arm B) as pre-intervention treatment for patients with advanced/metastatic renal cell carcinoma who are eligible for cytoreductive nephrectomy, metastasectomy or repeat biopsy. Each patient in both Arms A and B will be given the anti-CTLA-4 monoclonal antibody tremelimumab at 10mg/kg every month for 2 doses prior to surgery or biopsy.

Patients in Arm B will additionally receive cryoablation of a metastasis prior to the first dose of tremelimumab. Cytoreductive nephrectomy, metastasectomy or repeat biopsy will occur 4-6 weeks after the 2nd dose of tremelimumab. The rationale is that tumor antigen release from cryoablation may prime T cell activation, which can augment tremelimumab efficacy and therefore, results in increased immune response against renal cell carcinoma. The primary objectives are to assess the safety and tolerability of tremelimumab alone and in combination with cryoablation. Secondary objectives include gathering initial estimates of immunological and molecular changes in tumor tissues and peripheral blood, performing neoantigen discovery on tumor tissue, and studying objective response rates and progression-free survival (PFS) experiences between the 2 arms. The purpose of randomization is to identify potential toxicity differences when combining these 2 therapies in comparison to tremelimumab alone.

7.1.1 Primary Endpoint: Safety

Safety will be recorded through the incidence of adverse events, serious adverse events and specific laboratory abnormalities (worst grade) in each treatment arm. Toxicities will be graded using the National Cancer Institute (NCI) Common Terminology Criteria for Adverse Events (CTCAE) version 4.03. For trial monitoring and decisions about future trials, extreme toxicities (TOX) will be defined as any grade 3 or higher adverse event that is possibly, probably, or definitely related to therapy that occurs within the first two cycles of therapy with the following exceptions: 1) Any grade 3 or higher adverse event that is potentially treatable with steroids will only count as an extreme toxicity if it does not improve to grade 1 or better within 2 weeks of steroid therapy. 2) Grade 3 or 4 amylase or lipase abnormalities that are not associated with symptoms or clinical manifestations of pancreatitis. 3) Grade 3 or 4 drug related endocrinopathies which within two weeks of presentation are adequately controlled with only physiologic hormone replacement therapy.

7.1.2 Secondary Endpoints

7.1.2.1. Immunological and molecular tests

Immunological variables will be measured based on peripheral blood (pb) samples and tumor tissue samples. Measurements based on blood samples will be made on weeks -4, 0, 1, 4, 8, 12, 16 and measurements from the tumor tissue at week 9.

7.1.2.2 Objective Response Rate

Objective response rate (ORR) is defined as the number of subjects with a best response of CR or PR by irRC (Table 5) criteria divided by the number of randomized subjects as described in Section 7.4

7.1.2.3 Progression-Free Survival (PFS)

PFS is defined as the time from randomization to the first documented tumor progression as determined by the investigator using RECIST 1.1 criteria or death due to any cause, whichever occurs first. Patients who are alive and free of known progression at the time of analysis will be censored on the date of last tumor assessment.

7.2 Sample Size

Up to 30 patients will be enrolled, for 15 patients in each arm. This sample size is based on having sufficient patients to assess toxicities in each arm while providing preliminary comparisons of immunology and secondary endpoints for future trials in patients with advanced renal cell carcinoma. With 15 patients in each arm, for safety, either arm is designed to stop early 19% of the time if the true toxicity rate is 25%, only 1% if the rate is as low as 10% and 58% if it's 40% (see safety monitoring below).

7.3 Randomization

Patients will be stratified by whether the patient is planned to have 1) cytoreductive nephrectomy, 2) metastasectomy, or 3) post-treatment biopsy and then randomized 1:1 through the Clinical Trial Conduct website (https://biostatistics.mdanderson.org/ClinicalTrialConduct), which is housed on a secure server at MDACC and maintained by the MDACC Department of Biostatistics. Access to the website will be gained through usernames and passwords provided by the MDACC Department of Biostatistics to personnel responsible for enrolling patients. Training on the use of the Clinical Trial Conduct website to enroll patients on the study will be provided by the study biostatistician for study personnel.

7.4 Safety Monitoring

All patients who receive at least one dose of tremelimumab or undergo cryoablation will be considered evaluable for toxicity. Based on the method of Thall et al[42] continual monitoring after the 5th patient is planned. Calculations were performed in Multc Lean. Denote the probability of TOX by θ_T, where TOX is defined in section 7.1.1 above. Since our definition of TOX is different from preliminary data available for tremelimumab or other anti-CTLA4 agents, for our prior, we will not use the prior data for the monitoring rule. Our stopping rule is given by the following probability statement: $Pr(\theta_T > 0.25 \mid data) > 0.85$. That is, we will stop the trial if, at any time during the study, we determine that there is more than an 85% chance that the TOX rate is more than 25%. Multc Lean version 1.2 was used to determine the stopping rules assuming a prior for "standard therapy" $\theta_T \sim$ beta (5, 15) and a prior $\theta_T \sim$ beta (0.50, 1.5) for the current study. The stopping boundaries for this toxicity rule are to terminate the trial if the number of patients with TOX compared to the number of patients on trial exceeds the limits in Table 6, with the operating characteristics for this rule in Table 7.

Table 6. Stopping Criteria for Excessive Toxicities based on TOX in Each Arm

If there are this many patients with TOX	3	4	5	6	7
Stop if this many patients (or fewer) have been evaluated at least once for toxicity	5	8	11	13	15

Table 7. The Operating Characteristics for Toxicity Monitoring Each Arm

True toxicity rate	Probability of Stopping Early	Median (25th %ile, 75th %ile)	Average Number of Patients	Average Number Patients with TOX
0.10	0.01	15 (15, 15)	14.9	1.5
0.20	0.10	15 (15, 15)	14.2	2.8
0.25	0.19	15 (15, 15)	13.5	3.4
0.30	0.31	15 (10, 15)	12.5	3.8
0.40	0.58	11 (5, 15)	10.3	4.1
0.50	0.81	5 (5, 11)	8.2	4.1

7.5 Data Analyses

Descriptive statistical analyses will be performed to summarize the overall TOX rate and individual adverse event rates, response rates, as well as immunological changes, including summary tables, scatter-plots, box-plots, proportions, 95% credible intervals, median, means, and standard deviations. The randomization stratification is for balancing purposes only. Strata with sufficient patients may be reported separately as an exploratory analysis. Differences of indication markers between arms will be compared using a t-test with transformations of non-normal data, as needed. PFS will be estimated with the methods of [43]. A mixed model accounting for patient effects will be used to analyze the longitudinal data on immunological values over time. The models will include treatment parameters representing the effects of the on weeks 1 and 4. For each of immunologic factors, denote the vector of longitudinal measurements by Z={Z(-4),Z(1),Z(3),Z(5), Z(7),Z(9),Z(11)} and the tumor measurement at week 13 by W. Possible relationships between immunologic measure Z(t) and W for each immunological variable will be assessed once the form of the population mean pathway for Z(t) has been determined. For example, if f(t) is linear then immunological response may be characterized by the slope of the line, and possible relationships between this slope and tumor response W may be assessed. Once the best longitudinal model has been identified, then the treatment arm will be added to the model and tested whether cryoablation is associated with the week 13 tumor measurement.

8.0 DATA AND PROTOCOL MANAGEMENT

8.1 Registration Procedure and Data Reporting

All patients will be registered in the approved Office of Research Administration database at MD Anderson Cancer Center such as Clinical Oncology Research System (CORe). Data will be entered into MD Anderson institutionally approved and compliant database(s). The database(s) have secure portal that requires users to login with validated credentials, uses approved encryption protocols as defined by institutional information security standards. Systems have granular data access controls to ensure that minimal amount of information required to complete a task is presented, can

handle de-linking and de-identification of patient information to maintain patient confidentiality if necessary. The system(s) are 21 CFR 11 compliant. Standard data collection, storage procedures, and quality assurance procedures will be followed, to ensure integrity and auditability of all information entered. Electronic case report forms for this trial will be in Prometheus. Reporting to the supporting agency will follow the contract agreement.

8.2 Clinical Trial Posting

Information related to this study will be posted on www.clinicaltrials.gov before the first patient is enrolled in the study.

9.0 ADVERSE EVENT REPORTING

9.1 Serious Adverse Event Reporting (SAE)

An adverse event or suspected adverse reaction is considered "serious" if, in the view of the investigator, it results in any of the following outcomes:

- Death
- A life-threatening adverse drug experience – any adverse experience that places the patient, in the view of the initial reporter, at immediate risk of death from the adverse experience as it occurred. It does not include an adverse experience that, had it occurred in a more severe form, might have caused death.
- Inpatient hospitalization or prolongation of existing hospitalization
- A persistent or significant incapacity or substantial disruption of the ability to conduct normal life functions.
- A congenital anomaly/birth defect.

- Important medical events that may not result in death, be life-threatening, or require hospitalization may be considered a serious adverse drug experience when, based upon appropriate medical judgment, they may jeopardize the patient or subject and may require medical or surgical intervention to prevent one of the outcomes listed in this definition. Examples of such medical events include allergic bronchospasm requiring intensive treatment in an emergency room or at home, blood dyscrasias or convulsions that do not result in inpatient hospitalization, or the development of drug dependency or drug abuse (21 CFR 312.32).

- Important medical events as defined above, may also be considered serious adverse events. Any important medical event can and should be reported as an SAE if deemed appropriate by the Principal Investigator or the IND Sponsor, IND Office.

- All events occurring during the conduct of a protocol and meeting the definition of a SAE must be reported to the IRB in accordance with the timeframes and procedures outlined in "The University of Texas M. D. Anderson Cancer Center Institutional Review Board Policy for Investigators on Reporting Unanticipated Adverse Events for Drugs and Devices". Unless stated otherwise in the protocol, all SAEs, expected

or unexpected, must be reported to the IND Office, regardless of attribution (within 5 working days of knowledge of the event).

- All life-threatening or fatal events, that are unexpected, and related to the study drug, must have a written report submitted within 24 hours (next working day) of knowledge of the event to the Safety Project Manager in the IND Office.
- Unless otherwise noted, the electronic SAE application (eSAE) will be utilized for safety reporting to the IND Office and MDACC IRB.
- Serious adverse events will be captured from the time of the first protocol-specific intervention, until 30 days after the last dose of drug, unless the participant withdraws consent. Serious adverse events must be followed until clinical recovery is complete and laboratory tests have returned to baseline, progression of the event has stabilized, or there has been acceptable resolution of the event.
- Additionally, any serious adverse events that occur after the 30 day time period that are related to the study treatment must be reported to the IND Office. This may include the development of a secondary malignancy.

Reporting to FDA:

- Serious adverse events will be forwarded to FDA by the IND Sponsor (Safety Project Manager IND Office) according to 21 CFR 312.32.

- It is the responsibility of the PI and the research team to ensure serious adverse events are reported according to the Code of Federal Regulations, Good Clinical Practices, the protocol guidelines, the sponsor's guidelines, and Institutional Review Board policy.

9.2 Reporting of Adverse Events

Adverse Events will be documented according to the Recommended Adverse Event Recording Guidelines (see Table 8 below).

Table 8. Recommended Adverse Event Recording Guidelines

**Recommended Adverse Event Recording Guidelines**						
**Attribution**	**Grade 1**	**Grade 2**	**Grade 3**		**Grade 4**	**Grade 5**
**Unrelated**	Phase I	Phase I	Phase I<br>Phase II		Phase I<br>Phase II<br>Phase III	Phase I<br>Phase II<br>Phase III
**Unlikely**	Phase I	Phase I	Phase I<br>Phase II		Phase I<br>Phase II<br>Phase III	Phase I<br>Phase II<br>Phase III
**Possible**	Phase I<br>Phase II	Phase I<br>Phase II<br>Phase III	Phase I<br>Phase II<br>Phase III		Phase I<br>Phase II<br>Phase III	Phase I<br>Phase II<br>Phase III
**Probable**	Phase I<br>Phase II	Phase I<br>Phase II	Phase I<br>Phase II		Phase I<br>Phase II	Phase I<br>Phase II

		Phase III	Phase III		Phase III	Phase III
**Definitive**	Phase I<br>Phase II	Phase I<br>Phase II<br>Phase III	Phase I<br>Phase II<br>Phase III		Phase I<br>Phase II<br>Phase III	Phase I<br>Phase II<br>Phase III

The Investigator or physician designee will be responsible for verifying and providing source documentation for all adverse events and assigning the attribution for all subjects enrolled on the trial.

9.3 Reporting Requirements to Supporting Company:

- For studies conducted under an IND, any event that is both serious and unexpected must be reported to the FDA as soon as possible and, in no event, later than 7 days (death or life-threatening event) or 15 days (all other SAEs) after the investigator's or institution's initial receipt of the information. Medimmune will be provided with a simultaneous copy of all adverse events filed with the FDA. SAEs will be reported on the MDACC SAE Form.

- All serious, unexpected, related SAEs should be e-mailed to Medimmune Patient Safety at the same time the report is sent to the FDA, at: AEMailboxClinicalTrialTCS@astrazeneca.com
Monthly, and not later than 15 calendar days after the end of each calendar month to enable MedImmune to meet its regulatory obligations.

- The investigator is responsible for obtaining follow-up information which becomes available as the SAE evolves, as well as supporting documentation (e.g., hospital discharge summaries and autopsy reports). The investigator is responsible for updating the SAE report and providing the follow-up SAE report(s) to MedImmune Patient Safety using the same procedure as the initial SAE report.

- AEs should be followed to resolution or stabilization, and reported as SAEs if they become serious. This also applies to subjects experiencing AEs that cause interruption or discontinuation of tremelimumab, or those experiencing AEs that are present at the end of their participation in the study; such subjects should receive post-treatment follow-up as appropriate.

- In Medimmune supported trials, all SAEs must be collected which occur within 70 days of discontinuation of dosing or completion of the patient's participation in the study if the last scheduled visit occurs at a later time. In addition, if at anytime after 70 days since last dose of study drug the Investigator becomes aware of an SAE and assesses it as certainly, probably, or possibly related to tremelimumab, MedImmune should be notified as above.

Notes:

- Cancer/Overdose: An overdose is defined as the accidental or intentional ingestion of any dose of a product that is considered both excessive and medically

important. For reporting purposes, Medimmune considers an overdose, regardless of adverse outcome, as an important medical event. All cases of cancer and overdose must be reported immediately to Medimmune.

- Hospitalizations (exceptions): Criteria for hospitalizations not reported as SAEs include admissions for:
  - Planned as per protocol medical/surgical procedure
  - Routine health assessment requiring admission for baseline/trending of health status documentation (e.g., routine colonoscopy)
  - Medical/surgical admission for purpose other than remedying ill health state (planned prior to entry into study trial; appropriate documentation required)
  - Admission encountered for other life circumstance that carries no bearing on health status and requires no medical/surgical intervention (e.g. lack of housing, economic inadequacy, care-giver respite, family circumstances, administrative)

An SAE report should be completed for any event where doubt exists regarding its status of seriousness.

9.4 Pregnancy

Patients must agree to use adequate contraception (barrier method of birth control) 28 days prior to first dose of study drug and up to 180 days after the last dose of study drug. Should a patient's sexual partner become pregnant or suspect she is pregnant while the patient is participating in this study, he should inform the treating physician immediately.

9.5 Tremelimumab adverse events of special interest

An adverse event of special interest (AESI) is one of scientific and medical interest specific to understanding of the Investigational Product and may require close monitoring and rapid communication by the investigator to the sponsor. An AESI may be serious or non-serious. The rapid reporting of AESIs allows ongoing surveillance of these events in order to characterize and understand them in association with the use of this investigational product.

AESIs for tremelimumab include but are not limited to events with a potential inflammatory or immune-mediated mechanism and which may require more frequent monitoring and/or interventions such as steroids, immunosuppressants and/or hormone replacement therapy. These AESIs are being closely monitored in clinical studies with tremelimumab monotherapy and combination therapy. An immune-related adverse event (irAE) is defined as an adverse event that is associated with drug exposure and is consistent with an immune-mediated mechanism of action and where there is no clear alternate aetiology. Serologic, immunologic, and histologic (biopsy) data, as appropriate, should be used to support an irAE diagnosis. Appropriate efforts should be made to rule out neoplastic, infectious, metabolic, toxin, or other etiologic causes of the irAE.
If the Investigator has any questions in regards to an adverse event (AE) being an

irAE, the Investigator should promptly contact the Study Physician.
AESIs observed with tremelimumab include:

- Colitis
- Pneumonitis
- ALT/AST increases / hepatitis / hepatotoxicity
- Neuropathy / neuromuscular toxicity (i.e. events of encephalitis, peripheral motor and sensory neuropathies, Guillain-Barré, and myasthenia gravis)
- Endocrinopathy (i.e. events of hypophysitis, adrenal insufficiency, and hyper- and hypothyroidism)
- Dermatitis
- Nephritis
- Pancreatitis (or labs suggestive of pancreatitis - increased serum lipase , increased serum amylase)

Further information on these risks (e.g. presenting symptoms) can be found in the current version of the durvalumab and tremelimumab Investigator Brochure. For tremelimumab, AESIs will comprise the following:

Pneumonitis

AEs of pneumonitis are also of interest for AstraZeneca, as pneumonitis has been observed with use of anti-PD-1 mAbs (but not with anti-PD-L1 mAbs). Initial work-up should include a high-resolution CT scan, ruling out infection, and pulse oximetry. Pulmonary consultation is highly recommended. Guidelines for the management of patients with immune-related AEs (irAEs) including pneumonitis are provided in Appendix F Table 1.

Infusion reactions

AEs of infusion reactions (also termed infusion-related reactions) are of special interest to AstraZeneca and are defined, for the purpose of this protocol, as all AEs occurring from the start of IP infusion up to 48 hours after the infusion start time. For all infusion reactions, SAEs should be reported to AstraZeneca Patient safety.

Hypersensitivity reactions

Hypersensitivity reactions as well as infusion-related reactions have been reported with anti-PD-L1 and anti-PD-1 therapy (Brahmer et al 2012) As with the administration of any foreign protein and/or other biologic agents, reactions following the infusion of mAbs can be caused by various mechanisms, including acute anaphylactic (IgE-mediated) and anaphylactoid reactions against the mAbs and serum sickness. Acute allergic reactions may occur, may be severe, and may result in death. Acute allergic reactions may include hypotension, dyspnea, cyanosis, respiratory failure, urticaria, pruritus, angioedema, hypotonia, arthralgia, bronchospasm, wheeze, cough, dizziness, fatigue, headache, hypertension, myalgia, vomiting, and unresponsiveness. Guidelines for the management of patients with hypersensitivity (including anaphylactic reaction) and infusion-related reactions are provided in Appendix F Table 1.

Hepatic function abnormalities (hepatotoxicity)

Hepatic function abnormality is defined as any increase in ALT or AST to greater than 3 × ULN and concurrent increase in total bilirubin to be greater than 2 × ULN.

Concurrent findings are those that derive from a single blood draw or from separate blood draws taken within 8 days of each other. Follow-up investigations and inquiries will be initiated promptly by the investigational site to determine whether the findings are reproducible and/or whether there is objective evidence that clearly supports causation by a disease (eg, cholelithiasis and bile duct obstruction with distended gallbladder) or an agent other than the IP. Guidelines for management of patients with hepatic function abnormality are provided in (Appendix F Table1)

Gastrointestinal disorders

Diarrhea/colitis is the most commonly observed treatment emergent SAE when tremelimumab is used as monotherapy. In rare cases, colon perforation may occur that requires surgery (colectomy) or can lead to a fatal outcome if not properly managed. Guidelines on management of diarrhea and colitis in patients receiving tremelimumab are provided in Appendix F Table 1.

Endocrine disorders

Immune-mediated endocrinopathies include hypophysitis, adrenal insufficiency, and hyper- and hypothyroidism. Guidelines for the management of patients with immune-mediated endocrine events are provided in Appendix F Table 1.

Pancreatic disorders

Immune-mediated pancreatitis includes autoimmune pancreatitis, and lipase and amylase elevation. Guidelines for the management of patients with immune-mediated pancreatic disorders are provided in Appendix F Table 1.

Neurotoxicity

Immune-mediated nervous system events include encephalitis, peripheral motor and sensory neuropathies, Guillain-Barré, and myasthenia gravis. Guidelines for the management of patients with immune-mediated neurotoxic events are provided in Appendix F Table 1.

Nephritis

Consult with Nephrologist. Monitor for signs and symptoms that may be related to changes in renal function (e.g. routine urinalysis, elevated serum BUN and creatinine, decreased creatinine clearance, electrolyte imbalance, decrease in urine output, proteinuria, etc)

Patients should be thoroughly evaluated to rule out any alternative etiology (e.g., disease progression, infections etc.)

Steroids should be considered in the absence of clear alternative etiology even for low grade events (Grade 2), in order to prevent potential progression to higher grade event. Guidelines for the management of patients with immune-mediated neurotoxic events are provided in Appendix F Table 1.

10.0 REFERENCES

1. Krummel MF, Allison JP. CD28 and CTLA-4 have opposing effects on the response of T cells to stimulation. *J Exp Med.* Aug 1 1995;182(2):459-465.
2. Walunas TL, Bakker CY, Bluestone JA. CTLA-4 ligation blocks CD28-dependent T cell activation. *J Exp Med.* 1996;183:2541-2550.
3. Read S, Malmstrom V, Powrie F. Cytotoxic T lymphocyte-associated antigen 4 plays an essential role in the function of CD25(+)CD4(+) regulatory cells that control intestinal inflammation. *J Exp Med.* 2000;192(2):295-302.
4. Salomon B, Lenschow DJ, Rhee L, et al. B7/CD28 costimulation is essential for the homeostasis of the CD4+CD25+ immunoregulatory T cells that control autoimmune diabetes. *Immunity.* Apr 2000;12(4):431-440.
5. Brunner MC, Chambers CA, Chan FK, Hanke J, Winoto A, Allison JP. CTLA-4-Mediated inhibition of early events of T cell proliferation. *J Immunol.* May 15 1999;162(10):5813-5820.
6. Karandikar NJ, Vanderlugt CL, Walunas TL, Miller SD, Bluestone JA. CTLA-4: A negative regulator of autoimmune disease. *J.Exp.Med.* 1996;184:783-788.
7. Leach DR, Callahan GN. Fibrosarcoma cells expressing allogeneic MHC Class II antigens induce protective antitumor immunity. *J.Immunol.* 1995;154:738-143.
8. van Elsas A, Hurwitz AA, Allison JP. Combination immunotherapy of B16 melanoma using anti-cytotoxic T lymphocyte-associated antigen 4 (CTLA-4) and granulocyte/macrophage colony-stimulating factor (GM-CSF)-producing vaccines induces rejection of subcutaneous and metastatic tumors accompanied by autoimmune depigmentation. *J Exp Med.* Aug 2 1999;190(3):355-366.
9. Hodi FS, O'Day SJ, McDermott DF, et al. Improved survival with ipilimumab in patients with metastatic melanoma. *N Engl J Med.* Aug 19 2010;363(8):711-723.
10. Robert C, Thomas L, Bondarenko I, et al. Ipilimumab plus dacarbazine for previously untreated metastatic melanoma. *N Engl J Med.* Jun 30 2011;364(26):2517-2526.
11. Ribas A, Kefford R, Marshall MA, et al. Phase III randomized clinical trial comparing tremelimumab with standard-of-care chemotherapy in patients with advanced melanoma. *J Clin Oncol.* Feb 10 2013 2013;31(5):616-622.
12. Sangro B, Gomez-Martin C, de la Mata M, et al. A clinical trial of CTLA-4 blockade with tremelimumab in patients with hepatocellular carcinoma and chronic hepatitis C. *J Hepatol.* Mar 4 2013 2013.
13. Tarhini AA. Tremelimumab: a review of development to date in solid tumors. *Immunotherapy.* Mar 2013 2013;5(3):215-229.
14. Tarhini AA, Cherian J, Moschos SJ, et al. Safety and efficacy of combination immunotherapy with interferon alfa-2b and tremelimumab in patients with stage IV melanoma. *J Clin Oncol.* Jan 20 2012;30(3):322-328; 2012.
15. Vonderheide RH, LoRusso PM, Khalil M, et al. Tremelimumab in combination with exemestane in patients with advanced breast cancer and treatment-associated modulation of inducible costimulator expression on patient T cells. *Clin Cancer Res.* Jul 1 2010;16(13):3485-3494; 2010.
16. Rini BI, Stein M, Shannon P, et al. Phase I dose-escalation trial of tremelimumab plus sunitinib in patients with metastatic renal cell carcinoma. *Cancer* Feb 15 2011; 117(4):758-767; 2011
17. Jemal A, Siegel R, Ward E, et al. Cancer statistics, 2008. *CA: a Cancer Journal for Clinicians.* 2008;58(2):71-96.
18. Uzzo RG, Novick AC. Nephron sparing surgery for renal tumors: indications, techniques

and outcomes. *Journal of Urology.* 2001;166(1):6-18.

19. Ahrar K, Matin S, Wood CG, et al. Percutaneous radiofrequency ablation of renal tumors: technique, complications, and outcomes. *Journal of Vascular & Interventional Radiology.* 2005;16(5):679-688.

20. Bang HJ, Littrup PJ, Goodrich DJ, et al. Percutaneous cryoablation of metastatic renal cell carcinoma for local tumor control: feasibility, outcomes, and estimated cost-effectiveness for palliation. *J Vasc Interv Radiol.* Jun 2012 2012;23(6):770-777.

21. Callstrom MR, Dupuy DE, Solomon SB, et al. Percutaneous image-guided cryoablation of painful metastases involving bone: multicenter trial. *Cancer.* 2013;119(5):1033-1041.

22. Dupuy DE, Liu D, Hartfeil D, et al. Percutaneous radiofrequency ablation of painful osseous metastases: a multicenter American College of Radiology Imaging Network trial. *Cancer.* Feb 15 2010 2010;116(4):989-997.

23. Goetz MP, Callstrom MR, Charboneau JW, et al. Percutaneous image-guided radiofrequency ablation of painful metastases involving bone: a multicenter study. *J Clin Oncol.* Jan 15 2004;22(2):300-306.

24. McMenomy BP, Kurup AN, Johnson GB, et al. Percutaneous cryoablation of musculoskeletal oligometastatic disease for complete remission. *J Vasc Interv Radiol.* Feb 2013 2013;24(2):207-213.

25. Ahrar K, Wallace MJ, Matin SF. Percutaneous radiofrequency ablation: minimally invasive therapy for renal tumors. *Expert Rev Anticancer Ther.* 2006 Dec 2006;6(12).

26. Motzer RJ, Hutson TE, Tomczak P, et al. Overall survival and updated results for sunitinib compared with interferon alfa in patients with metastatic renal cell carcinoma. *J Clin Oncol.* Aug 1 2009;27(22):3584-3590.

27. Matin SF, Sharma P, Gill IS, et al. Immunological response to renal cryoablation in an in vivo orthotopic renal cell carcinoma murine model. *J Urol.* Jan 2010;183(1):333-338.

28. Sabel MS. Cryo-immunology: a review of the literature and propesd mechanisms for stimulatory versus supressive immune responses. *Cryobiology.* 2009;58:1-11.

29. Sabel MS, Arora A, Su G, Chang AE. Adoptive immunotherapy of breast cancer with lymph node cells primed by cryoablation of the primary tumor. *Cryobiology.* 2006;53(3):360-366.

30. Gursel E, Roberts, M., Veenema, R.J. e. Regression of Prostatic Cancer Following Sequential Cryotherapy to the Prostate. *1972.* 1972;108:928-932.

31 Neel HB, Ketcham, A.S., Hammond, W.G. ExperimentalEvaluation of In-Situ Oncocide for Primary Tumour Therapy: Comparison of Tumor Specific Immunity After Complete Excision, Cryonecrosis, and Ligation. *Laryngoscope.* 1973;83:376-387.

32. Soanes WA, Ablin, R.J., Gonder, M.J. Remission of Metastatic Lesions Following Cryosurgery in Prostatic Cancer: Immunologic Considerations. *J. Urology.* 1970;104:154-159.

33. Sanchez-Ortiz RF, Tannir N, Ahrar K, Wood CG. Spontaneous regression of pulmonary metastases from renal cell carcinoma after radiofrequency ablation of the primary tumor: an in situ tumor vaccine? *In press.* 2003.

34. Waitz R, Solomon SB, Petre EN, et al. Potent induction of tumor immunity by combining tumor cryoablation with anti-CTLA-4 therapy. *Cancer Res.* Jan 15 2012;72(2):430-439.

35. Casal RF, Tam AL, Eapen GA. Radiofrequency ablation of lung tumors. *Clin Chest Med.* Mar 2010;31(1):151-163, Table of Contents.

36. Karam JA, Ahrar K, Wood CG, et al. Radio frequency ablation of renal tumors in patients with metastatic renal cell carcinoma. *J Urol.* Nov 2010;184(5):1882-1887.

37. Tam A, Ahrar K. Palliative interventions for pain in cancer patients. *Semin Intervent Radiol.*

Dec 2007;24(4):419-429.
38. Arlen PM, Madan RA, Hodge JW, Schlom J, Gulley JL. Combining Vaccines with Conventional Therapies for Cancer. *Update Cancer Ther.* Mar 2007;2(1):33-39.
39. Drake CG. Combination immunotherapy approaches. *Ann Oncol.* Sep 2012;23 Suppl 8:viii41-46.
40. Maio M, Di Giacomo AM, Robert C, Eggermont AM. Update on the role of ipilimumab in melanoma and first data on new combination therapies. *Curr Opin Oncol.* Mar 2013;25(2):166-172.
41. Wolchok JD, Hoos A, O'Day S, et al. Guidelines for the evaluation of immune therapy activity in solid tumors: immune-related response criteria. *Clin Cancer Res.* Dec 1 2009;15(23):7412-7420.
42. Thall PF, Simon RM, Estey EH. Bayesian sequential monitoring designs for single-arm clinical trials with multiple outcomes. *Stat Med.* Feb 28 1995;14(4):357-379.
43. Kaplan EL, Meier P. Nonparametric estimation from incomplete observations. *Journal of the American Statistical Association.* 1958;53:457-481.
44. Wolchok J, Hoos A, O'Day S, et al. Guidelines for the evaluation of immune therapy activity in solid tumors: immune-related response criteria. *Clin Cancer Res.* 2009;15(23):7412-7419.
45. Liakou, C.I., et al., *CTLA-4 blockade increases IFNg-producing CD4+ICOShi cells to shift the ratio of effector to regulatory T cells in cancer patients.* Proc Natl Acad Sci USA, 2008. 105(39): p. 14987-14992.
46. Carthon, B.C., et al., *Preoperative CTLA-4 blockade: tolerability and immune monitoring in the setting of a presurgical clinical trial.* Clin Cancer Res, 2010. 16(10): p. 2861-71.
47. Tang, D.N., et al., *Increased Frequency of ICOS+ CD4 T Cells as a Pharmacodynamic Biomarker for Anti-CTLA-4 Therapy* Cancer Immunol Res, 2013. 1(4): p. 229–34.
48. Chen, H., et al., *CD4 T Cells Require ICOS-Mediated PI3K Signaling to Increase T-Bet Expression in the Setting of Anti-CTLA-4 Therapy.* Cancer Immunol Res, 2014. 2(2): p. 1-10.
