# Supplementary material for: Pilot study of Tremelimumab with and without cryoablation in patients with metastatic renal cell carcinoma
Source: Nat Commun. 2021 Nov 4;12:6375. doi: 10.1038/s41467-021-26415-4 (PMC8569213; doi:10.1038/s41467-021-26415-4)
Supplement: Supplementary file 2 — Reporting Summary [file 41467_2021_26415_MOESM2_ESM.pdf]

nature portfolio

Corresponding author(s): Padmanee Sharma

Last updated by author(s): 2021/09/28

Reporting Summary

Nature Portfolio wishes to improve the reproducibility of the work that we publish. This form provides structure for consistency and transparency in reporting. For further information on Nature Portfolio policies, see our Editorial Policies and the Editorial Policy Checklist.

Statistics

For all statistical analyses, confirm that the following items are present in the figure legend, table legend, main text, or Methods section.

n/a	Confirmed	
☐	☒	The exact sample size (*n*) for each experimental group/condition, given as a discrete number and unit of measurement
☐	☒	A statement on whether measurements were taken from distinct samples or whether the same sample was measured repeatedly
☐	☒	The statistical test(s) used AND whether they are one- or two-sided<br>*Only common tests should be described solely by name; describe more complex techniques in the Methods section.*
☐	☒	A description of all covariates tested
☐	☒	A description of any assumptions or corrections, such as tests of normality and adjustment for multiple comparisons
☐	☒	A full description of the statistical parameters including central tendency (e.g. means) or other basic estimates (e.g. regression coefficient) AND variation (e.g. standard deviation) or associated estimates of uncertainty (e.g. confidence intervals)
☐	☒	For null hypothesis testing, the test statistic (e.g. *F*, *t*, *r*) with confidence intervals, effect sizes, degrees of freedom and *P* value noted<br>*Give P values as exact values whenever suitable.*
☒	☐	For Bayesian analysis, information on the choice of priors and Markov chain Monte Carlo settings
☐	☒	For hierarchical and complex designs, identification of the appropriate level for tests and full reporting of outcomes
☐	☒	Estimates of effect sizes (e.g. Cohen's *d*, Pearson's *r*), indicating how they were calculated

Our web collection on statistics for biologists contains articles on many of the points above.

Software and code

Policy information about availability of computer code

Data collection	Aperio ImageScope, HALO 2.3.2089.70, Prism 8.4.3 GraphPad, nSolver 4.0 and advanced analysis 2.0 NanoString, QJucore 3.7
Data analysis	Aperio ImageScope, HALO 2.3.2089.70, Prism 8.4.3 GraphPad, nSolver 4.0 and advanced analysis 2.0 NanoString, Qlucore 3.7, SASv9.4, Stata 16.1

For manuscripts utilizing custom algorithms or software that are central to the research but not yet described in published literature, software must be made available to editors and reviewers. We strongly encourage code deposition in a community repository (e.g. GitHub). See the Nature Portfolio guidelines for submitting code & software for further information.

Data

Policy information about availability of data

All manuscripts must include a data availability statement. This statement should provide the following information, where applicable:

- Accession codes, unique identifiers, or web links for publicly available datasets
- A description of any restrictions on data availability
- For clinical datasets or third party data, please ensure that the statement adheres to our policy

The authors declare that the data supporting the findings in this study are available in the manuscript and its supplementary information and source data files. The NanoString data that support the findings of this study are available as Supplementary Data 1 (normalized Log2 counts) with the manuscript. Other relevant data related to the current study will be available from the corresponding author (P.S.) based upon a reasonable academic request and will require a data access agreement with the University of Texas at MD Anderson Cancer Center and the requester as the information may include data collected under an institutional alliance clinical trial protocol. Source data and clinical trial protocol are provided as Source data and Supplementary Note 1 respectively.

Field-specific reporting

Please select the one below that is the best fit for your research. If you are not sure, read the appropriate sections before making your selection.

[x] Life sciences [ ] Behavioural & social sciences [ ] Ecological, evolutionary & environmental sciences

For a reference copy of the document with all sections, see nature.com/documents/nr-reporting-summary-flat.pdf

Life sciences study design

All studies must disclose on these points even when the disclosure is negative.

Sample size	Sample size was selected to ensure that safety of the combination of cryoablation and tremelimumab could be tested as compared to tremelimumab alone. The sample sizes were sufficient because they enabled us to test our hypothesis and observe statistically significant differences. The sample size of the study was 30 participants. A Bayesian design was used to determine sample size, the rationale for the sample size was provided in the protocol in section 7.2, 7.4, table 6 and table 7. "With 15 patients in each arm, for safety, either arm is designed to stop early 19% of the time if the true toxicity rate is 25%, only 1% if the rate is as low as 10% and 58% if it's 40%." Fifteen patients was selected in each arm based on modeling.
Data exclusions	No data was excluded
Replication	Since the studies were performed on patient tissue samples replication of each sample was not feasible due to limited amount of sample.
Randomization	Patients were randomized 1:1 to receive cryoablation plus tremelimumab vs tremelimumab alone. No stratification was used on the study.
Blinding	Blinding for patients was not possible as we did not find it ethical to perform a shame procedure for cryoablation. Blinding for providers was not performed. Given the small sample size and the pilot nature of the study we did not feel it was necessary to blind the investigators of the arm assigned to the study participants.

Reporting for specific materials, systems and methods

We require information from authors about some types of materials, experimental systems and methods used in many studies. Here, indicate whether each material, system or method listed is relevant to your study. If you are not sure if a list item applies to your research, read the appropriate section before selecting a response.

Materials & experimental systems

n/a	Involved in the study	
[ ]	[x]	Antibodies
[x]	[ ]	Eukaryotic cell lines
[x]	[ ]	Palaeontology and archaeology
[x]	[ ]	Animals and other organisms
[ ]	[x]	Human research participants
[ ]	[x]	Clinical data
[x]	[ ]	Dual use research of concern

Methods

n/a	Involved in the study	
[x]	[ ]	ChIP-seq
[x]	[ ]	Flow cytometry
[x]	[ ]	MRI-based neuroimaging

Antibodies

Antibodies used	CD3 (Agilent Dako, cat#A045 2, clone F7 .2.38 ,1:100), CD20 (Agilent Dako, cat#M0755 01-2, clone L26,1:1400), CDS (Thermo Scientific, cat# MS-457-S, clone CS/144B,1:25), Granzyme B (Leica Microsystems, cat# PA0291, clone llFl,RTU) and PD-1 (Abeam, cat# ab137132, clone EPR4877,1:250)
Validation	Each Primary antibody was validated based on manufacturer's instructions. https://www.agilent.com/en/product/immunohistochemistry/antibodies-controls/primary-antibodies/cd3-(concentrate);https://www.agilent.com/en/product/immunohistochemistry/antibodies-controls/primary-antibodies/cd20cy-(concentrate)-76520,https://assets.thermofisher.com/TFS-Assets/APD/Specification-Sheets/D11869.pdf, https://shop.leicabiosystems.com/us/ihc-ish/ihc-primary-antibodies/pid-granzyme-b,https://www.abcam.com/pd1-antibody-epr48772-ab137132.html

Human research participants

Policy information about studies involving human research participants

Population characteristics	32 patients with metastatic renal cell carcinoma with a lesion that was considered eligible for cryoablation were enrolled. Table 1 provides baseline demographics for the enrolled patients.
Recruitment	Patients with metastatic renal cell carcinoma at MD Anderson Cancer Center were approached for consideration of trial participation and if they agreed they signed consent and went through screening to ensure they meet inclusion and exclusion criteria. We used available tumor samples from the patients enrolled in the trial. There was no potential self selection bias.
Ethics oversight	The study was approved by The University of Texas MD Anderson Cancer Center 's IND office with oversight by our Institutional Review Board (IRB), Immunotherapy Platform umbrella protocol PA13-0291. The study was compliant with all relevant regulations pertaining to the use of human study participants and was conducted in accordance with the criteria set by the Declaration of Helsinki.

Note that full information on the approval of the study protocol must also be provided in the manuscript.

Clinical data

Policy information about clinical studies

All manuscripts should comply with the ICMJEguidelines for publication of clinical research and a completedCONSORT checklist must be included with all submissions.

Clinical trial registration	NCT02626130
Study protocol	The protocol is uploaded as Supplementary Notes 1 and information regarding the protocol is also available at clinicaltrials.gov (NCT02626130).
Data collection	The patients were enrolled at University of Texas MD Anderson Cancer Center at Houston Texas. The study was activated in 2014 and the recruitment started on October 31st 2016 and ended on October 25th 2018.All clinical data from the study was collected in Prometheus, software developed at MD Anderson Cancer Center and Microsoft excel spreadsheets. Data was collected from 2016 through July 2021
Outcomes	Patient and tumor characteristics were tabulated at the time of study entry. This trial was a randomized pilot study to investigate the safety of adding cryoablation to tremelimumab therapy among patients undergoing surgery or repeat biopsy for metastatic renal cell carcinoma (mRCC). The primary outcome was safety. The primary endpoint of the study was safety using the National Cancer Institute (NCI) Common Terminology Criteria for Adverse Events (CTCAE) version 4.0. Each arm was followed for rate of extreme toxicity which was defined as any grade 3 or higher adverse event that did not improve in severity within two weeks of corticosteroid therapy. The safety monitoring was continuous after the 5th patient was enrolled with strict stopping rules if extreme toxicity was found at an unacceptable rate in either arm. Secondary outcomes included clinical response, progression-free (PFS) and overall survival (OS) were reported but not designed or powered for comparison between the two arms. Patients were classified as having response if best RECIST assessment over the course of the study was partial response (PR) or complete response (CR) and clinical benefit if their response was CR, PR, or stable disease (SD). Differences in proportions were tested with exact chi-square tests. Overall survival (OS) was defined as the number of months from randomization to death (event) or last contact for patients who were alive at the final data collection. Progression-free survival (PFS) was defined as the time from randomization until progression or death, whichever came first (event) or last follow-up for disease assessment among patients who were alive and free-of disease at the last assessment. PFS and OS were calculated and graphed by Kaplan-Meier methods. Differences were tested by log-rank test. Adverse events were tabulated overall and by treatment arm among events that occur within either arm. Each patient counted once at the highest grade experienced for each event. Related events were events that were ever classified as being possibly, probably, or definitely related to study therapy. All analyses were performed in SAS 9.4 [SAS Institute Inc., Cary, NC] and figures were generated in Stata 14 [StataCorp LLC, College Station, TX].
