# Supplementary material for: Pilot study of Tremelimumab with and without cryoablation in patients with metastatic renal cell carcinoma
Source: Nat Commun. 2021 Nov 4;12:6375. doi: 10.1038/s41467-021-26415-4 (PMC8569213; doi:10.1038/s41467-021-26415-4)
Supplement: Supplementary file 3 — Descriptions of Additional Supplementary Files [file 41467_2021_26415_MOESM3_ESM.docx]

**Supplementary Data 1**. NanoString dataset of log2 normalized counts obtained from pre-and post-treatment tissue samples of metastatic renal cell carcinoma patients enrolled in the trial. Patients were treated with tremelimumab monotherapy or cryoablation plus tremelimumab combination therapy.
